# Activity‐Enhancing Mutations in an LmrR‐Based Artificial Metalloenzyme Destabilize the Protein Scaffold and Alter its Conformational Plasticity

**DOI:** 10.1002/cbic.202500259

**Published:** 2025-07-09

**Authors:** Adil A. Safeer, Fabrizio Casilli, Wouter Beugelink, Gerard Roelfes, Marc Baldus, Hugo van Ingen

**Affiliations:** ^1^ NMR Spectroscopy Bijvoet Center for Biomolecular Research Utrecht University Padualaan 8 3584 CH Utrecht The Netherlands; ^2^ Biomolecular Chemistry & Catalysis Stratingh Institute for Chemistry University of Groningen Nijenborgh 3 9747 AG Groningen The Netherlands; ^3^ Structural Biochemistry Bijvoet Center for Biomolecular Research Utrecht University Universiteitsweg 99 3584 CG Utrecht The Netherlands

**Keywords:** artificial metalloenzymes, biocatalyses, enzyme design, nuclear magnetic resonance, protein dynamics

## Abstract

Artificial metalloenzymes (ArM) hold great potential for the sustainable catalysis of complex new‐to‐nature reactions. To efficiently improve the catalytic efficacy of ArMs, a rational approach is desirable, requiring detailed molecular insight into their conformational landscape. Lactococcal multidrug resistance regulator (LmrR) is a multipurpose ArM scaffold protein that, when bound to the Cu(II)‐phenanthroline cofactor, catalyzes the Friedel–Crafts alkylation (FCA) of indoles. Previously, the M8D and A92E mutations are found to increase the efficiency of this reaction, but a molecular explanation has been lacking. The impact of these two activating mutations on the conformational landscape of LmrR in its apo, cofactor‐ and substrate‐bound states is determined. The mutations cause a marked destabilization of the dimerization interface, resulting in a more open central hydrophobic cavity and a dynamic equilibrium between dimer and monomer LmrR is found. While mutant and wild‐type have similar pocket conformation in the cofactor‐bound state, the mutant shows a distinct interaction with the substrate. Our results suggest that increased retention of the catalytic cofactor and widened plasticity improve the activity of the mutant. Ultimately, these results help elucidating the intricate relationships between conformational dynamics of the protein scaffold, cofactor, and substrates that define catalytic activity.

## Graphical abstract

The protein LmrR is a versatile scaffold to design artificial metalloenzymes. Here, we show that the M8D/A92E (DE) mutations that result in a much more efficient catalyst, destabilize the protein dimerization interface and alter the conformation landscape when binding metal cofactor and substrates are shown. These results highlight the intricate relations between protein, metal cofactor, and substrates in defining catalytic efficiency.

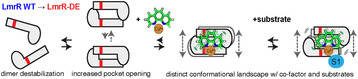

## 1 Introduction

Green chemical synthesis requires the efficient catalysis of reactions that are new‐to‐nature. In particular, artificial metalloenzymes (ArM) expand the scope of possible chemical reactions and offer opportunities to design an appropriate protein scaffold around a transition metal of interest.^[^ ^1^, ^2^, ^3^, ^4^ ^]^ A particularly versatile protein scaffold is the lactococcal multidrug resistance regulator (LmrR) derived from *Lactococcus lactis*.^[^ ^5^, ^6^ ^]^ LmrR has been successfully applied in the catalysis of the Friedel–Crafts alkylation (FCA) of indoles using noncovalently bound Cu(II)‐phenanthroline (Cu(II)‐Phen) as the catalytic cofactor.^[^ ^7^, ^8^ ^]^ In addition, LmrR has been used catalyze the Diels–Alder reaction of azachalcone with cyclopentadiene, both using Cu(II)‐Phen and through covalent cysteine‐linkage of a Cu(II)‐bipyridine complex,^[^ ^7^, ^8^, ^9^ ^]^ as well as oxime or hydrazone formation via the introduction of a noncanonical amino acid.^[^ ^10^, ^11^, ^12^ ^]^

To optimize its catalytic efficacy, LmrR has been subjected to directed evolution and rational protein design approaches.^[^ ^7^, ^8^ ^]^ However, it remains challenging to create highly active “designer enzymes”. It is becoming increasingly clear that rational enzyme design should not only consider the static 3D structures of enzymes but also their conformational dynamics.^[^ ^13^, ^14^, ^15^, ^16^ ^]^ Solution nuclear magnetic resonance (NMR) spectroscopy can provide a wealth of information on the conformational landscapes of proteins and enzymes^[^ ^17^, ^18^, ^19^ ^]^ contribution. In the context of protein engineering, NMR has been used to reveal depopulation of inactive conformations in directed evolution^[^ ^20^ ^]^ and to guide directed evolution by identifying key residues in transition‐state stabilization.^[^ ^21^ ^]^

**Figure 1:**
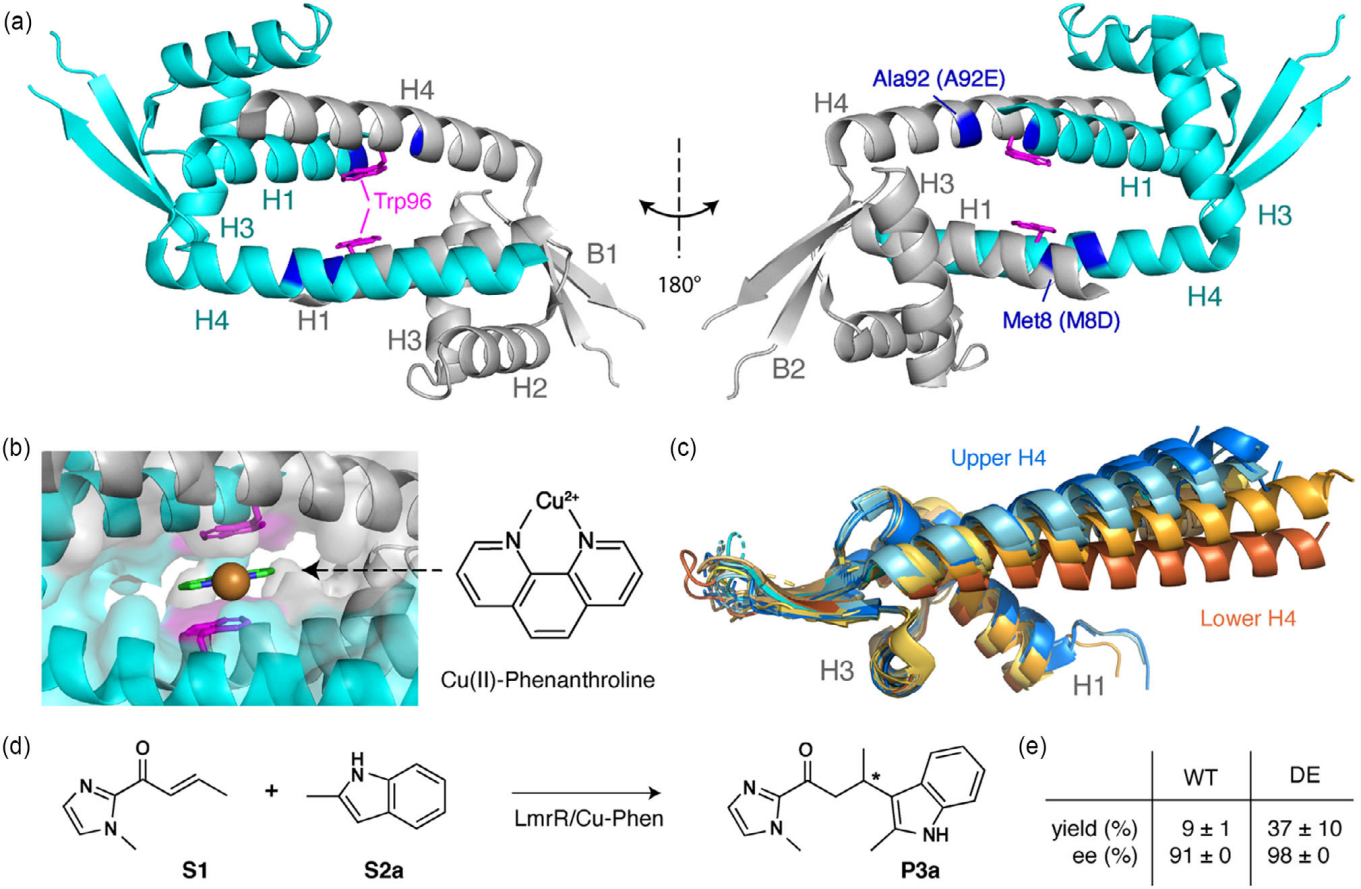
Structures of LmrR. a) LmrR homodimer structure (PDB: 6R1L) with two chains colored in gray and cyan. The DE mutation sites and ligand‐coordinating Trp96 residues are indicated. b) Zoom‐in on the hydrophobic pocket in complex with the Cu(II)‐phenanthroline (Cu(II)‐Phen) cofactor (PDB: 6R1L). Molecular structure of Cu(II)‐Phen is shown. c) Superposition of LmrR structures with various ligands, highlighting the upper (blue tints) and lower (brown tints) conformations of H4. Full details can be found in Table S1, Supporting Information. d) FCA reaction scheme of substrates S1 (α,β‐unsaturated 2‐acyl‐1‐methylimidazole) and S2a (2‐methylindole) to form product P3a. Stereospecific center indicated with *. e) Yield and enantiomeric selectivity (ee) for a 6 h reaction as in d) for WT and DE‐mutant LmrR (12 μM) with 9 μM Cu(II)‐Phen and 1 mM substrates in 20 mM MOPS, 150 mM NaCl, pH 7, 4 °C.^[^ ^7^ ^]^

LmrR is known to be an intrinsically dynamic protein that is thought to employ an allosteric regulation mechanism for DNA transcription in *L*. *lactis*.^[^ ^22^, ^23^, ^24^ ^]^ The protein is comprised of a homodimer in which the DNA binding domains (DBDs) of each monomer are joined by a central helical bundle (**Figure** **1**a). This helical bundle, formed by helix H1, H3, and H4, can bind a variety of compounds in its central cavity through planar π‐stacking interactions with the Trp96 indoles from both monomers (Figure 1b).^[^ ^8^, ^22^, ^25^, ^26^, ^27^, ^28^, ^29^ ^]^ A comparison of various LmrR crystal structures shows considerable conformational heterogeneity in the relative orientation of helix H4 (Figure 1c). This orientation varies between an “upper” and “lower” conformation, which are coupled to an expanded, respectively contracted distance between the H3 helices.^[^ ^22^, ^23^, ^24^ ^]^ Previously, Takeuchi et al.^[^ ^23^ ^]^ used a series of solution NMR experiments to find that in solution, the protein is in a dynamic equilibrium between the H4 upper and lower conformations, and that binding of compounds in the central pocket shifts the equilibrium toward the upper conformation.

In the catalysis of the FCA reaction, the LmrR ArM is formed by coordination of Cu(II)‐Phen in the central cavity via π‐stacking interactions with Trp96. Previously, two mutations in this cavity, M8D and A92E, were found to considerably increase the yield and enantioselectivity of the FCA of indoles (see Figure 1d,e).^[^ ^7^, ^8^ ^]^ Molecular dynamics simulations^[^ ^8^, ^29^, ^30^ ^]^ suggested that the A92E mutation promoted a more favorable arrangement of the Trp96 sidechains, allowing for stronger cofactor binding compared to the wild‐type (WT). In the case of the double mutant (M8D/A92E, abbreviated as DE), binding of Cu(II)‐Phen has been found to be ≈150‐fold stronger than the parent.^[^ ^7^, ^8^ ^]^ Thus, the DE mutant could be expected to enforce even better positioning of the cofactor. The proximity of the DE mutations in the 3D structure can, however, be expected to generate electrostatic repulsion in the hydrophobic pocket (Figure 1b), which may alter the conformational equilibria of LmrR, and thereby influence catalytic efficiency.

Here, we used solution NMR spectroscopy to compare the structural dynamics of the WT and the double mutant (LmrR‐DE) in the apo state, in complex with the transition metal cofactor Zn(II)‐phenanthroline as a diamagnetic analog of Cu(II)‐Phen, and in the presence of reaction substrates. Our data show that the mutations destabilize the dimer interface in the apo state, resulting in a monomer–dimer equilibrium, and favor an open pocket state. In the cofactor‐bound state, the conformational dynamics of the pocket and the cofactor itself are similar in WT and DE mutant, while there is a striking difference in interaction with substrate. Together, our results help to rationalize the increased binding affinity of the cofactor for the mutant and the conformational dynamics in both apo and cofactor‐bound LmrR.

## 2 Results and Discussion

### 2.1 LmrR‐DE Mutations Destabilize the Dimer Interface

Both WT and DE LmrR variants show relatively well‐dispersed NMR spectra in the apo state, indicating that both proteins were well‐folded (Figure S1 and S2, Supporting Information). In total, 83% of the WT LmrR backbone resonances and 75% of the DE variant could be assigned. Line broadening and extensive overlap prevented complete backbone assignment with most unassigned residues in α‐helices H1 and H4. Comparison of backbone amide chemical shifts reveals that the largest changes due to the mutations occur in α‐helices H3 and H4, around the DBD, suggestive of subtle differences in helical packing between WT and the mutant (Figure S3, Supporting Information).

**Figure 2:**
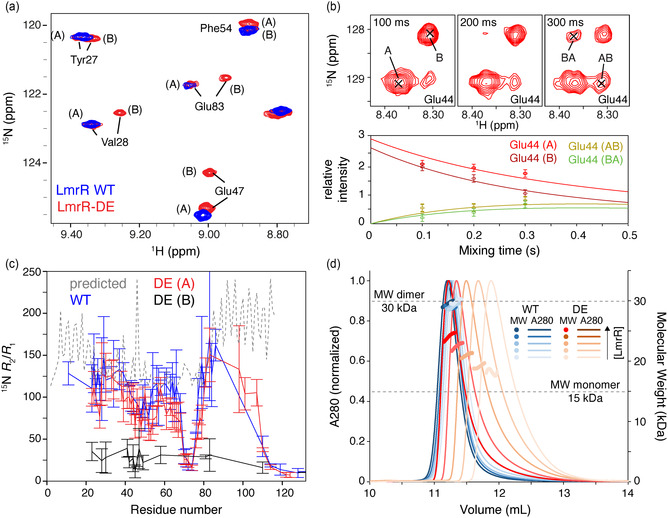
The apo LmrR‐DE mutant exists in a dynamic equilibrium between dimer and monomer. a) Section of ^15^N‐HSQC spectra showing peak duplicity for LmrR‐DE, with dimer peaks labeled “A” and monomer labeled “B”. b) Section of ^15^N ZZ‐exchange spectra showing cross peaks between state “A” and “B” resonances of Glu44 (top) together with fit of the extracted intensities of diagonal (A, B) and cross peaks (AB, BA) to a 1:1 exchange model. c) Backbone ^15^N *R*_2_/*R*_*1*_ relaxation rate ratio per residue for monomer/dimer mutant and WT LmrR compared to structure‐based predicted rates using HydroNMR.^[^ ^41^ ^]^ d) Absorbance at 280 nm and MW of WT and mutant LmrR as a function of protein concentration as tracked by SEC‐MALS. Color coding is indicated in the figure.

Interestingly, for several residues of LmrR‐DE duplicate amide backbone correlations could be observed. Some can be attributed to cis/trans proline isomerization in the unstructured termini of LmrR. However, for several residues in the DBD, two clear peaks denoted as states “A” and “B” could be observed, while this was not the case for the WT where only the peaks overlapping with state “A” were detected (**Figure** **2**a). ZZ‐exchange experiments revealed a dynamic interconversion between the two states with a global exchange rate of 3.1 s^−1^ (Figure 2b), as probed for residues Glu42, Glu44, and Thr49. Interestingly, the fractional population of state “B” decreased with increasing LmrR concentrations (Figure S4, Supporting Information).

To further understand the origin of the state “B” peaks, ^15^N backbone relaxation experiments were performed to determine the transversal (*R*_2_) and longitudinal (*R*_1_) relaxation rates. Analysis of the *R*_2_/*R*_1_ values, proportional to correlation time (*τ*_c_), and thus, the overall molecular dimensions, showed that the WT and mutant state “A” LmrR behave mostly in line with predicted relaxation rates based on the structure (PDB: 3F8F) (Figure 2c). Strikingly, the *R*_2_/*R*_1_ values for state “B” resonances of the LmrR‐DE residues are half that of state “A” resonances, indicating that state “B” belongs to monomeric LmrR with half of the *τ*_c_ and half the size of the dimer, state “A”.

Further analysis using size‐exclusion chromatography multiangle light scattering (SEC‐MALS) experiments confirmed the presence of monomeric species in the DE variant. The average mass of the DE variant decreases with protein concentration, from ≈30 kDa corresponding to the molecular weight (MW) of an LmrR dimer to ≈17 kDa, whereas this effect is not seen for the WT (Figure 2d, Table S2, Supporting Information). Based on the NMR dilution series, the dissociation constant (*K*_D_) for the dimerization equilibrium equals ≈33 μM (see Table S2, Supporting Information).

Notably, the predicted secondary structure based on the Cα/Cβ chemical shifts of the monomer peaks indicates that monomer residues retain a similar secondary structure as in the dimer (Figure S6, Supporting Information). Although we cannot exclude that H4 is partly unfolded in the monomer due to the lack of resonance assignments in H4, we conclude that the monomer remains a folded species. This is also consistent with the similar melting temperatures between mutant (*T*_m_ = 51 °C, Figure S7, Supporting Information) and WT (*T*_m_ = 50 °C).^[^ ^31^ ^]^

Together, these data show that the DE mutations destabilize the dimer interface, resulting in an observable dynamic equilibrium between dimer and monomer species. This likely results from electrostatic repulsion between the spatially close Asp8 and Glu92 residues.

### 2.2 The Mutant Hydrophobic Pocket is More Open in the Apo State

As the DE mutations are in the H1/H4 interface, we next wanted to probe to what extent the H4 upper/lower conformation was affected. Previously, Takeuchi et al. identified the gauche‐ and trans‐rotameric states of residue Ile62 as a critical reporter of this equilibrium.^[^ ^23^ ^]^ Conveniently, the Ile62 δ1 methyl ^13^C chemical shift is correlated to the population of the trans and gauche‐rotamers along the χ_2_ angle.^[^ ^32^ ^]^ According to this method, the trans‐ and gauche‐rotameric states are fully occupied at 14.8 and 9.3 ppm, respectively.^[^ ^32^ ^]^ Thus, the ^13^C δ1 chemical shift of Ile62 can be used to directly probe the H4 conformational state.

**Figure 3:**
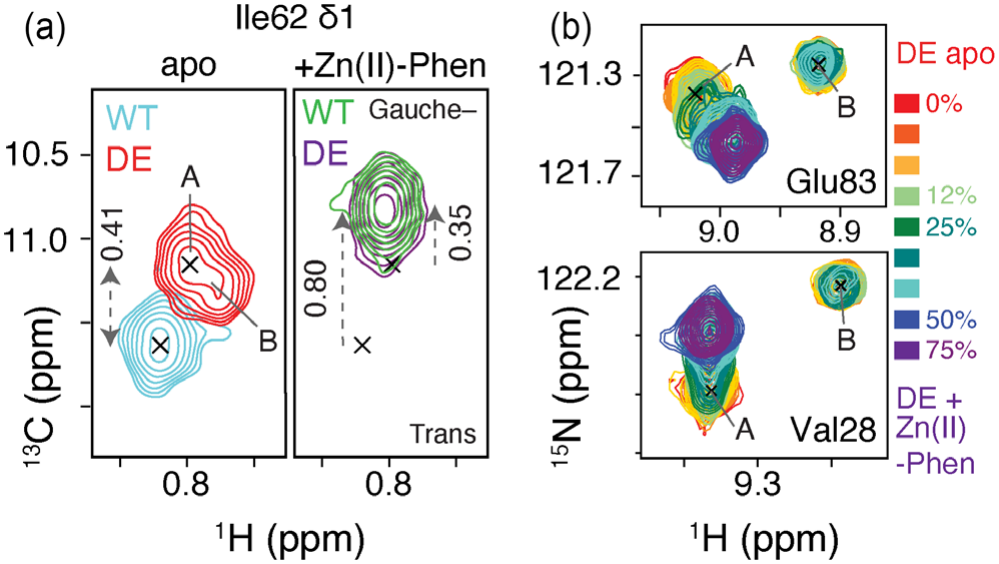
Comparison of hinge residue Ile62 conformation and stabilization of mutant dimer upon cofactor binding. a) Apo LmrR‐DE has an upfield‐shifted Ile62 δ1 resonance compared to WT LmrR, corresponding to increased population of Ile62 gauche and H4 upper conformation (left). Binding of Zn(II)‐Phen cofactor results in the same conformation for WT and mutant (right). b) Titration of Zn(II)‐Phen induces peak movement of dimer resonance (A) and eventual disappearance of monomer resonance (B). Color coding is indicated in the figure.

Notably, two peaks can be observed for the Ile62 δ1 resonance in apo LmrR‐DE, labeled “A” and “B” in **Figure** **3**a. We attribute the state “B” peak to Ile62 in the monomer state, see also below. Comparison of the WT and DE variants showed distinct ^13^C Ile62 δ1 chemical shifts in the apo state, at 298 K (Figure 3a). The observed chemical shift difference of 0.4 ppm translates into an increased population of the H4 upper state for LmrR‐DE compared to WT, with a *p*_g‐_ (population gauche‐rotamer) of 65% for DE versus 57% for the WT.

Previous structural analysis of three LmrR structures correlated the H4 conformation to the distance between the H3 helices.^[^ ^22^, ^23^ ^]^ Expansion of this analysis to eight currently available crystal structures of WT LmrR (Table S1, Supporting Information) confirmed this correlation and further identified a weaker correlation to the distance between the H1 helices (Figure S8, Supporting Information). Since the H1/H1' and H3/H3' distances define the dimension on one side of the pocket (see Figure 1b and S8, Supporting Information), structures with the upper H4 orientation also tend to have an expanded, larger pocket.

Thus, the data above indicate that the repulsion introduced by the DE mutations on one hand destabilizes the dimer, creating a significant population of monomer LmrR and on the other hand alters the helical configuration within the LmrR dimer, increasing the H4 upper conformation and widening the hydrophobic pocket in its apo state. These effects combined likely cause the increased binding affinity for the cofactor. On one hand, the Gibbs free energy (G) of apo LmrR‐DE is raised compared to WT and will, thus, contribute to a larger *ΔG* for cofactor binding. On the other hand, the widened pocket may more readily bind the cofactor.

### 2.3 Cofactor Binding Stabilizes the LmrR Dimer and Increases Pocket Opening

To study the effect of cofactor binding, we used the less reactive diamagnetic Zn(II)‐based cofactor (see Figure S9, Supporting Information) to circumvent signal line broadening and attenuation induced by paramagnetic relaxation effects from the Cu(II)‐Phen cofactor. Titration of Zn(II)‐Phen to the protein pushed the monomer–dimer equilibrium of LmrR‐DE in the direction of dimerization as the monomer backbone signals (states “B”) were attenuated and eventually disappeared upon saturation with cofactor (Figure 3b). Similarly, the state “B” peak for the Ile62 δ1 in the mutant disappeared upon cofactor titration (see Figure 3c). This indicates that the cofactor binding energy is more than sufficient to offset the dimer interface destabilization due to the DE mutation. Notably, the cofactor not only “glue” the two monomers together through the aromatic π–π stacking but also contributes a + 2 charge that will compensate for the negative charge in the pocket.

Cofactor binding caused significant chemical shift perturbations (CSPs) mostly in H4 but also throughout the protein, highlighting the allosteric nature of the protein (Figure S10, Supporting Information). Interestingly, several peaks (such as for the amide resonance of Val28 in Figure 3b) show a pronounced movement toward the monomer peak position (marked “B”) of the apo state. Assuming that helix H4 is unrestrained in the monomer and can, thus, be considered in the “upper” conformation, this suggests that the H4 conformation is pushed to the upper conformation upon cofactor binding.

Indeed, addition of the Zn(II)‐Phen cofactor to LmrR resulted in an upfield peak shift of Ile62 δ1 for both WT and mutant (Figure 3c), indicating an increase in gauche‐rotamer population and increased population of the helix H4 upper conformation, in line with what was observed for other compounds.^[^ ^23^ ^]^ Notably, the cofactor‐bound states in both variants have nearly the same Ile62 δ1 chemical shift (*p*_g–_ 72%), and thus, contain open dimer cavities to a similar degree.

### 2.4 Altered Energetics of Pocket Dynamics in the DE‐Mutant

So far, our data have indicated that the DE mutation destabilizes the central cofactor binding pocket corresponding to a more open state of the pocket in the apo state, that binding energy from the cofactor compensates for the inherent instability, and that WT and mutant have a similar degree of pocket opening in the bound state, at least at 298 K. We next aimed to characterize the dynamic conformational equilibrium of the LmrR pocket in more detail.

Following the approach of Takeuchi et al.^[^ ^23^ ^]^ the temperature dependence of the Ile62 *χ*_2_ trans‐ and gauche‐rotamer population was determined from the Ile62 C_δ1_ chemical shift for both WT and DE‐mutant LmrR, in the apo and cofactor‐bound state. The resulting Van't Hoff plots were used to determine changes in the energetics of this conformational equilibrium. Notably, the Ile62 rotamer state is not directly affected by the DE mutations due to the distance from the two mutation sites. Rather, any impact on its conformational equilibrium is a result of the coupling between the helical packing of H1/H4, H1/H3, and H3/H4 (see Figure S8, Supporting Information), and thus, indirectly reports on the conformational state of the pocket.

**Figure 4:**
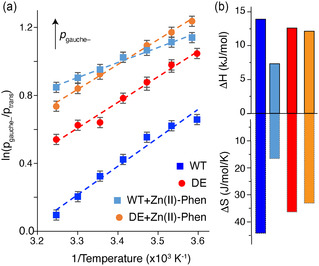
Thermodynamic analysis of the Ile62 *χ*_2_ rotamer equilibrium. a) Van't Hoff plot for WT and DE‐mutant LmrR in apo and Zn(II)‐Phen bound states. Dashed lines indicate best‐fit lines. Direction of increasing gauche‐population (*p*_gauche–_, corresponding to more open pocket) indicated. b). Extracted Δ*H*_trans‐gauche–_ and Δ*S*_trans‐gauche–_, reflecting the conversion from gauche to trans Ile62 *χ*_2_ rotamer, corresponding to the closing of the pocket. Color coding is indicated in the figure.

In all cases, the gauche‐rotamer population (corresponding to an opened pocket) increases with decreasing temperatures, indicating that the open state is enthalpically favored and dominant at low temperatures (**Figure** **4** and S11, Supporting Information), in line with previous work.^[^ ^23^ ^]^ The thermodynamic parameters extracted for WT LmrR (Δ*H*_trans‐gauche–_ and Δ*S*_trans‐gauche–_, corresponding to closing of the pocket) are also reasonably close to those determined by Takeuchi et al.^[^ ^23^ ^]^ (Δ*H*/Δ*S* = +13.9 kJ mol^−1^/+44 J mol^−1^ K^−1^ vs. Δ*H*/Δ*S* = +18.9 kJ mol^−1^/+62 J mol^−1^ K^−1^), given the differences in LmrR construct and buffer conditions.

In the apo state, the DE‐mutant has a more open pocket compared to WT at all temperatures (Figure 4b and S11, Supporting Information). On top of that, the DE variant has a reduced temperature‐dependent chemical shift range compared to WT, 0.59 versus 0.74 ppm, respectively (Figure S11, Supporting Information). Linear regression of the Van't Hoff plot data indicates that both Δ*H*_trans ‐ gauche–_ and Δ*S*_trans ‐ gauche–_ are smaller for DE compared to WT (10% for Δ*H* and 20% for Δ*S*).

The reduction in Δ*S* for apo DE is in agreement with the reduced hydrophobicity of the pocket upon the DE mutations. Compared to WT, there will be less entropic gain from releasing structured water molecules upon closing of the pocket, resulting in a lower Δ*S*_trans ‐ gauche–_ for DE. At 298 K, this entropic effect (≈2.3 kJ mol^−1^) dominates the enthalpic difference between WT and DE (1.3 kJ mol^−1^).

When the Zn(II)‐Phen cofactor is bound in the pocket, the impact of buried water can be expected to be reduced. Indeed, WT and DE mutants behave much more similar in the cofactor‐bound state (Figure 4). Still, three notable observations can be made: i) while Δ*H*_trans ‐ gauche–_ and Δ*S*_trans ‐ gauche–_ for the DE mutant remain quite similar in the bound state compared to the free state, there is an approximate twofold reduction in Δ*H* and Δ*S* for WT, resulting in a greater enthalpy–entropy compensation in the cofactor‐bound state of DE compared to WT (Figure 4b); ii) in the bound state, the conformational equilibrium is more temperature sensitive for the DE‐mutant compared to WT; and iii) at low temperatures the gauche‐(open) state is favored more in DE mutant than in WT, while at high temperatures the gauche‐(open) state is favored more in WT. This distinct behavior is, however, not readily correlated to the increase in activity, as the DE mutant is more active than WT at both 4 °C^[^ ^7^ ^]^ and 25 °C (Figure S9, Supporting Information). Thus, activity is not directly related to the degree of pocket opening, neither in the apo or cofactor‐bound state.

### 2.5 The Cofactor‐Bound Pocket Exhibits Significant Dynamics

Given the importance of the Zn(II)‐Phen cofactor, we next attempted to detect its NMR signals to obtain a more detailed understanding of its arrangement within the protein pocket. Notably, the diamagnetic Zn(II) cofactor is required here as the strong paramagnetic relaxation effect of Cu(II) would prevent observation of signals from nearby protons.

**Figure 5:**
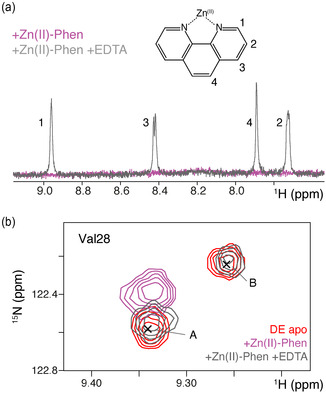
Stripping the Zn(II)‐ion with EDTA recovers the cofactor from the LmrR‐DE hydrophobic pocket. a) ^13^C/^15^N‐filtered 1D spectra of Zn(II)‐Phen bound LmrR‐DE with and without EDTA (1:1 molar ratio to dimer). Signals from the phenanthroline group are labeled. b) Comparison of backbone signal of Val28 in LmrR‐DE in apo and Zn(II)‐Phen bound with or without EDTA. Labels “A” and “B” refer to dimer and monomer peaks. Color coding is indicated in the figure.

To selectively detect the cofactor complexed with LmrR, ^13^C, ^15^N‐filtered 1D spectra were recorded on unlabeled cofactor‐bound to ^13^C, ^15^N‐labeled LmrR. Strikingly, no cofactor signals could be observed for both mutant (**Figure** **5**a, magenta) and WT (Figure S12, Supporting Information) LmrR, indicative of extensive line broadening. Similarly, also, in the presence of an overabundance of FCA substrate **S1** (α,β‐unsaturated 2‐acyl‐1‐methylimidazole) and **S2a** (2‐methylindole),^[^ ^7^ ^]^ no cofactor signal could be detected (Figure S12, Supporting Information).

The expected pattern of four cofactor signals appears when (EDTA) ethylenediaminetetraaetic acid is added in a 1:1 ratio to strip the Zn^2+^ ion from the phenanthroline (Figure 5a, gray). Strikingly, EDTA addition also causes the reappearance of monomer LmrR‐DE resonances, reversal of the dimer peak CSPs (Figure 5b), and return to the apo state value of the *p*_g‐_ of the Ile62 δ1 sensor (Figure S13, Supporting Information). These observations indicate that stripping the Zn‐ion from Zn(II)‐Phen also releases the phenanthroline from the pocket.

Thus, we conclude that there is considerable dynamics in the cofactor‐bound pocket, such that the cofactor can move relative to the coordinating Trp96 residues. The Trp96 aromatic rings will induce large aromatic ring current shifts that are highly dependent on the exact relative orientation of the cofactor and indole ring. Since the cofactor line broadening was observed for both WT and DE LmrR, this suggests that catalysis does not depend on a rigidly bound cofactor.

### 2.6 Substrate Presence Widens the Conformational Landscape of LmrR

We next asked whether interaction of the substrates of the FCA reaction with cofactor‐bound LmrR would further alter the protein conformational state differently for WT versus the DE variant. In the proposed reaction mechanism for a Cu(II)‐catalyzed FCA reaction, substrate **S1** is expected to coordinate the Cu(II)‐Phen, after which substrate **S2a** reacts with **S1**.^[^ ^33^ ^]^ However, no reaction is possible when only substrate **S1** or **S2a** is added.

By adding a high excess of substrate (7.5 mM), we maximized the population of the enzyme–cofactor–substrate complex to extract its NMR fingerprint. As the planar and aromatic FCA substrates could potentially compete with the Zn(II)‐Phen cofactor for the binding pocket at these high levels of excess, we first analyzed the pattern of amide backbone CSPs and the impact on the pocket from the Ile62 δ1 chemical shift (Figure S13, S14, Supporting Information). Addition of **S2a** caused significant CSPs for several residues, mostly in the DBD (Figure S14, Supporting Information), and decreased the Ile62 gauche‐population (Figure S13, Supporting Information). As this could signify that the indole **S2a** can substitute for the cofactor, these data were not further analyzed. In contrast, only small CSPs were observed upon **S1** addition (Figure S13, S14, Supporting Information), indicating that a high excess of **S1** does not outcompete the cofactor.

**Figure 6:**
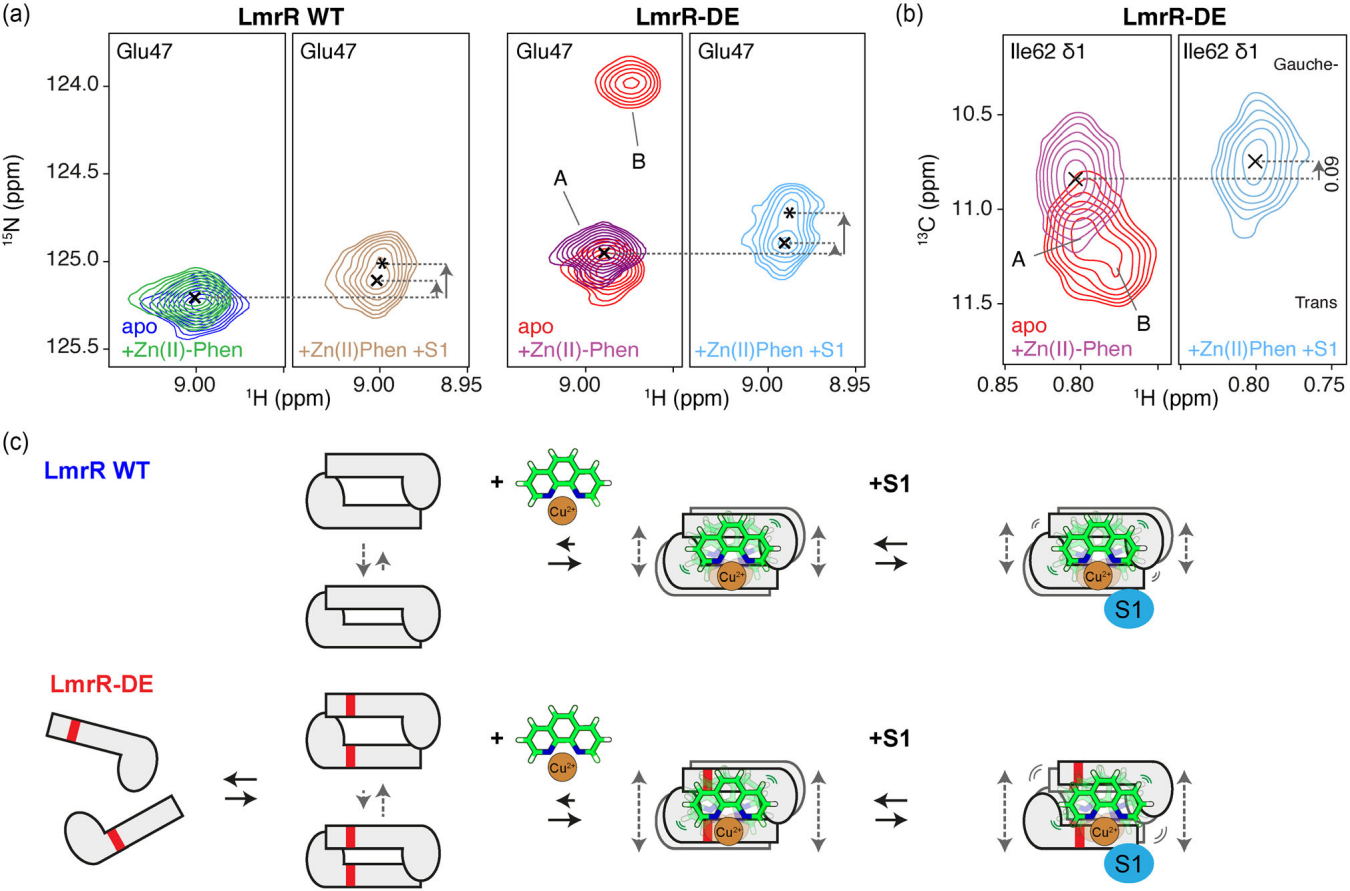
NMR‐based characterization of substrate binding to the LmrR–cofactor complex. a) The amide backbone peak for Glu47 shifts upon addition of (7.5 mM) substrate **S1** to the LmrR + Zn(II)‐Phen complex. The additional state is marked with *. Chemical shift changes in the ^15^N dimension are highlighted. Monomer and dimer resonance for the apo mutant are denoted with “A” and “B”. b) Additional Ile62 δ1 peak movement occurs upon substrate addition toward Ile62 gauche/more open pocket conformation. c) Schematic summary of main findings: the M8D and A92E mutations in LmrR‐DE (indicated as red bars) cause destabilization of protein dimer interface, resulting in monomeric LmrR species and increased pocket opening in the dimer (gray dashed arrows); Cu(II)‐phenanthroline binding stabilizes the dimer in an open pocket state with distinct thermodynamic profile in LmrR‐DE; addition of S1 results in increased pocket opening in LmrR‐DE with distinct conformational impact. Color coding is indicated in the figure.

Strikingly, the addition of **S1** makes new states appear for several LmrR residues (**Figure** **6**a and S15, Supporting Information). This effect is conserved for residues across the WT and DE variants of LmrR. The affected residues are scattered over the protein and may, therefore, report an allosteric effect. Close inspection shows that the peaks for the additional state are slightly more distinct for LmrR‐DE compared to WT, likely due to a slightly larger chemical shift difference between states and/or slower exchange between states (Figure 6a).

As the substrates can be expected to bind weakly to the enzyme–cofactor complex, the two signals cannot correspond to free enzyme–cofactor (E_C_) and enzyme–cofactor–substrate complex (E_C_S1) as those would be expected to be in fast exchange. Rather, we attribute the main peak to be the fast‐exchange average of E_C_ and E_C_S1, and the second peak to correspond to an alternative conformation of the E_C_S1 complex, E_C_S1*, that is in slow exchange with E_C_S1.

Notably, for several residues, the chemical shift of both E_C_S1 and E_C_S1* state is in the direction of the monomer resonance position present for apo LmrR‐DE, suggesting a further opening of the pocket in this state (Figure 6a and S15, Supporting Information). Indeed, we observe a slight upfield shift (0.09 ppm) of the Ile62 δ1 resonance upon **S1** binding (Figure 6b), corresponding to a slight increase of the Ile62 gauche‐state population (open pocket).

These results suggest that when **S1** is coordinated to the transition metal, the Cu(II)‐Phen–**S1** complex is bound further into the pocket, thus, creating a more open pocket. The data further suggest this involves a slow conformational transition that is more pronounced in the DE mutant compared to WT. Interestingly, in the study of the glycosidase BcX, a similar second state in slow exchange with the main enzyme‐substrate (ES) complex signals was observed, which was attributed to originate from an ES* complex in which the substrate is distorted.^[^ ^34^ ^]^ Since the mutated residues are far from the cofactor and the substrate binding site, a direct role in binding the cofactor or substrates seems unlikely. Rather, the different energetics of the conformational equilibria in the mutant may promote the required plasticity to permit effective coordination of the substrates.

### 2.7 Mutagenesis of Hotspot Residues

Finally, we examined all CSP data collected during this study to identify “hotspot” residues with clear CSPs upon DE mutation, cofactor binding, and substrate binding. In total, 12 residues were identified. These were all distant from the catalytic site, highlighting the allosteric coupling between the central cavity/dimer interface and the DBD. Mutagenesis in these suspected hotspots, however, failed to deliver an improved variant (Figure S16, Supporting Information), though not all possible amino acid variations were tested at the sites of interest. Interestingly, the additional F54L mutation in the core of DBD resulted in a variant (LmrR‐DLE) that was significantly less active than LmrR‐DE (39% vs. 69%). Although a detailed characterization of this mutant is beyond the scope of this work, this result underscores that there is a functional coupling between the DBD and the catalytic center.

## 3 Conclusion

We here examined the conformational landscape of WT LmrR and a M8D/A92E mutant that has improved efficiency in catalyzing FCA of indoles using Cu(II)‐Phen as catalytic cofactor. The mutations introduce two negative charges in the central cofactor binding pocket, causing severe destabilization of the dimer interface. This results in dynamic equilibrium between LmrR dimer and monomer species and an increase in the population of the open pocket conformation in the apo DE dimer (see summary in Figure 6c). Both effects likely contribute to a much higher cofactor binding affinity of the mutant compared to the parent. Despite this higher affinity, the catalytic cofactor still displays significant dynamics in the pocket, suggesting that efficient catalysis does not require a rigidly bound cofactor.

The striking differences observed in the apo state largely disappear in the cofactor‐bound state, as cofactor binding stabilizes the dimer interface and results in a similar degree of pocket opening in WT and mutant. Next to a distinct thermodynamic profile of the pocket conformational equilibrium, the mutant shows a different conformational response upon interaction with substrate, suggestive of increased plasticity to accommodate the cofactor–substrate complex. We speculate that the DE mutant combines increased retention of the catalytic cofactor with increased plasticity to boost catalysis. Our data highlight the need to consider protein, cofactor, and substrates together in the search for rational design of further improved LmrR variants.

## 4 Experimental Section

4.1

4.1.1

##### Protein Growth and Purification

Chemically competent Lemo21(DE3) *Escherichia coli* cells were transformed with LmrR WT, LmrR‐DE, or LmrR‐DLE variants with a C‐terminal strep‐tag in a pET17b vector plasmid. All three tested variants contained K55D and K59Q mutations that reduce binding to cellular DNA/RNA.^[^ ^7^ ^]^ One colony of transformed bacteria was picked and grown overnight in a Luria–Bertoni (LB) medium preculture containing 100 mg L^−1^ ampicillin and 35 mg L^−1^ chloramphenicol at 37 °C. Growth was then scaled up to a final isotope‐enriched M9 minimal medium culture containing 0.5 g L^−1^ ^15^NH_4_Cl, 2 g L^−1^ U‐^13^C_6_ glucose, additionally supplemented with 100 mg L^−1^ ampicillin, and 35 mg L^−1^ chloramphenicol. Protein expression was induced at an OD_600_ of 0.8–0.9 with 0.4 mM (IPTG) isopropyl β‐D‐1‐thiogalactopyranoside, and cells were grown overnight at 20 °C. Likewise, for perdeuterated protein^[^ ^35^ ^]^ used in triple resonance NMR experiments, M9 medium was prepared in D_2_O and supplemented with ^13^C_6_‐d_7_ glucose. Cultures were harvested by centrifugation at 6000 × *g* for 20 min. The pelleted cells were resuspended in lysis buffer (50 mM NaHPO_4_, pH 8.0, 150 mM NaCl) and stored at −20 °C. Following the thawing of the resuspended pellet, 10 cycles of sonication were performed by pulsing on and off for 10 s at 13 kHz. Subsequently, five cycles of a 20 G needle pass‐through were performed before lysates were spun down at 20⋅10^3 ^× *g* for 30 min. Clear lysate was then loaded onto a 5 mL Strep‐Tactin Superflow cartridge (IBA Lifesciences) preequilibrated with the lysis buffer. Sufficient wash with lysis buffer was followed by elution using 5 mM desthiobiotin in the lysis buffer. Subsequently, the protein was buffer exchanged and gel filtration was performed using a Superdex 75 (Cytiva) column in the reaction buffer (20 mM (MOPS) 3‐morpholinopropane‐1‐sulfonic acid, pH 7.0, 150 mM NaCl). Purity was analyzed using (SDS‐PAGE) sodium dodecylsulfate polyacrylamide gel‐electrophoresis, and protein concentration was determined via absorbance at 280 nm (ε: 25.44⋅10^3 ^M^−1 ^cm^−1^) using a NanoDrop One microvolume UV‐VIS spectrophotometer (Thermo Fisher Scientific). For our experiments, protein concentration was calculated using the MW of the LmrR monomer unit which was determined using the Expasy ProtParam tool (MW WT: 15044.94 Da; MW DE: 15086.88 Da; MW DLE: 15052.86 Da). Typical yields for LmrR were 25 mg of protein per liter of culture. Protein stocks were flash‐frozen and stored at −80 °C.

##### In Vitro Assembly of the Active Metalloenzyme

Zn(II)‐Phen is readily soluble in aqueous environments and was directly added in a 1:1 ratio to the protein dimer from a stock solution to the protein in the reaction buffer to create the functional metalloenzyme. For the assembly of the ArM with the Cu(II)‐Phen cofactor, firstly, 5 mM of Cu(II)‐Phen stock was prepared in (DMSO) dimethyl sulfoxide. Cofactor was then added to the protein in a 4:1 ratio in 50 mL (minimal 80 μg mL^−1^ protein) of reaction buffer and incubated at room temperature for one hour. Afterwards, samples were concentrated using a centrifugal concentrator (MW cutoff: 10 kDa) and buffer exchanged tenfold with reaction buffer to eliminate free Cu(II)‐Phen.

##### SEC‐MALS Experiment

SEC‐MALS was performed to assess the dimerization states of the LmrR WT and LmrR‐DE variants. Protein was injected at a concentration range of 0.5–8.0 mg mL^−1^ onto a Superdex 75 Increase column (Cytiva) preequilibrated with the reaction buffer. A miniDAWN TREOS detector (Wyatt) and RID‐10A refractive index monitor (Shimadzu) were employed in this setup, and ASTRA6 software was used to analyze the recorded data.

##### CD Spectroscopy Measurements

Circular dichroism (CD) spectroscopy experiments were performed with 4 μM of dimer LmrR in 300 μL buffer containing only 20 mM MOPS pH 7. The thermostability assay was performed from 10 to 90 °C with a 0.5 °C⋅min^−1^ temperature ramp while recording at a wavelength of 222 nm. A reference sample for heat treatment comparison was prepared by heating the respective protein at the same concentration up to 95 °C for 1.5–5 h to ensure denaturation.

##### NMR Spectroscopy Measurements

Measurements of protonated U‐^13^C, ^15^N‐LmrR were performed on an Avance NEO 900 MHz Bruker spectrometer with a 5 mm TCI cryo‐probe at 298 K. All samples were prepared in the reaction buffer with 10% D_2_O in 5 mm tubes. Typical sample concentrations were 75 μM LmrR monomer, with the exception of the assignment experiments, which were carried out at 0.5 mM protein concentration. All data were processed using TopSpin 4.1 (Bruker BioSpin) and analyzed using NMRFAM‐SPARKY.^[^ ^36^ ^]^ Resonance assignments for the WT and DE variants of LmrR were obtained using triple resonance SO‐FAST^[^ ^37^ ^]^ HNCA, HNCACB, and HN(CO)CACB experiments recorded using perdeuterated U‐^13^C, ^15^N‐LmrR. Secondary structure predictions based on the acquired resonance assignments were achieved using TALOS‐N.^[^ ^38^ ^]^

Titrations of Zn(II)‐Phen in a 0%–100% range, and 7.5 mM of substrates S1 (in reaction buffer) and S2a (in acetonitrile) to protonated or perdeuterated U‐^13^C, ^15^N‐LmrR were monitored through 2D ^1^H, ^15^N‐(TROSY) transverse‐relaxation optimized spectroscopy fingerprint spectra. Chemical shit perturbations (CSP or Δδ) were calculated from the chemical shift changes in ppm in the ^1^H and ^15^N dimension (Δδ_1H_, Δδ_15N_) as

(1) $$\text{Δδ}=\sqrt{{\left({\text{Δδ}}_{1H}\right)}^{2}+{\left(\frac{{\text{Δδ}}_{15N}}{6.51}\right)}^{2}}$$

A dilution series of LmrR‐DE at protein concentrations of 400, 200, 100, and 50 μM was performed to probe the concentration dependence of the minor state. The number of scans was increased fourfold between every dilution step from 8 to 512 to maintain a constant signal‐to‐noise ratio. The ZZ‐exchange experiment was conducted with mixing times of 0.1, 0.2, and 0.3 s. Peak volumes of the state‐A and state‐B diagonal peaks (*I*_AA_ and *I*_BB_) and of the two exchange peaks (*I*_AB_ and *I*_BA_) global fit parameters for Glu42, Glu44, and Thr49 were determined by fitting the peak shape to a Gaussian function using PINT^[^ ^39^ ^]^ and fit according to the following equations^[^ ^40^ ^]^ below (6‐11) using MATLAB R2016b (The Mathworks).

(2) $$\lambda_{1}=0.5\cdot (R_{1}^{\text{AA}}+k_{1}+R_{1}^{\text{BB}}+k_{2}+\sqrt{\left(\left(R_{1}^{\text{AA}}-R_{1}^{\text{BB}}+k_{1}-k_{2}\right)+4\cdot k_{1}\cdot k_{2}\right)})$$

(3) $$\lambda_{2}=0.5\cdot (R_{1}^{\text{AA}}+k_{1}+R_{1}^{\text{BB}}+k_{2}-\sqrt{\left(\left(\;R_{1}^{\text{AA}}-R_{1}^{\text{BB}}+k_{1}-k_{2}\right)+4\cdot k_{1}\cdot k_{2}\right)})$$

(4) $$I^{\text{AA}}=0.5\cdot I_{0}^{\text{AA}}\cdot \left(\left(\left(1+\frac{R_{1}^{\text{BB}}-R_{1}^{\text{AA}}+k_{2}-k_{1}}{\lambda_{1}-\lambda_{2}}\right)\cdot e^{-\lambda_{2}\cdot t}\right)+\left(\left(1-\frac{R_{1}^{\text{BB}}-R_{1}^{\text{AA}}+k_{2}-k_{1}}{\lambda_{1}-\lambda_{2}}\right)\cdot e^{-\lambda_{1}\cdot t}\right)\right)$$

(5) $$I^{\text{AB}}=I_{0}^{\text{AA}}\cdot \left(\frac{k_{2}}{\lambda_{1}-\lambda_{2}}\right)\cdot (e^{-\lambda_{2}\cdot t}-e^{-\lambda_{1}\cdot t})$$

(6) $$I^{\text{BA}}=I_{0}^{\text{BB}}\cdot \left(\frac{k_{1}}{\lambda_{1}-\lambda_{2}}\right)\cdot \left(e^{-\lambda_{2}\cdot t}-e^{-\lambda_{1}\cdot t}\right)$$

(7) $$I^{\text{BB}}=0.5\cdot I_{0}^{\text{BB}}\cdot \left(\left(\left(1-\frac{R_{1}^{\text{BB}}-R_{1}^{\text{AA}}+k_{2}-k_{1}}{\lambda_{1}-\lambda_{2}}\right)\cdot e^{-\lambda_{2}\cdot t}\right)+\left(\left(1+\frac{R_{1}^{\text{BB}}-R_{1}^{\text{AA}}+k_{2}-k_{1}}{\lambda_{1}-\lambda_{2}}\right)\cdot e^{-\lambda_{1}\cdot t}\right)\right)$$

where *R*_1_^AA^ and *R*_1_^BB^ are the longitudinal relaxation rates of the “A” or “B” state and *k*_1_/*k*_2_ represent the forward/backward reactions rates.

The ^15^N‐T_1_ and ^15^N‐T_2_ experiments were recorded for WT and DE variants of LmrR with a sample concentration of 100 μM. The relaxation delay values were 0.01, 0.2, 0.6, and 1.2 s for the T_1_‐experiment and 15.8, 31.7, 47.6, and 63.4 ms for the T_2_‐experiment. Data were processed using NMRPipe and further analyzed with PINT^[^ ^39^ ^]^ to determine peak intensities and fit the relaxation rates. Experimental rates were compared to rates predicted from the LmrR structure (PDB: 3F8F) using HydroNMR.^[^ ^41^ ^]^

The Ile62 rotameric equilibrium was analyzed by recording methyl‐TROSY experiments at 308, 303, 298, 293, 288, 283, and 278 K for apo protein variants and at 308, 298, and 288 K for Zn(II)‐Phen bound to DE variant. The NMR standard (DSS) 2,2‐dimethyl‐2‐silapentane‐5‐sulfonate dissolved in the reaction buffer was used as an external chemical shift reference. The population of the gauche‐rotameric state (*p*_g‐_) of Ile62 was determined using Equation (8).^[^ ^32^ ^]^

(8) $$p_{\text{gauche}–}=\frac{14.8\text{ppm}-\delta_{\text{obs}}}{5.5\text{ ppm}}$$

where δ_obs_ is the observed Ile62 δ1 ^13^C chemical shift. A linear regression was used to fit the temperature series data in the Van't Hoff plot, with error in chemical shifts estimated by dividing the linewidth by the signal‐to‐noise ratio.

To probe cofactor signals, ^13^C/^15^N double‐filtered experiments^[^ ^42^ ^]^ were recorded, including a 2D NOESY experiments with 100 ms NOE mixing time. EDTA was added in a 1:1 ratio the protein–cofactor complex from a 0.5 M EDTA pH 7.5 stock.

The Zn(II)‐Phen cofactor (1 mM in reaction buffer) characterization and resonance assignment were performed at 298 K on an Avance II 600 MHz Bruker spectrometer with a 5 mm room temperature probe and was based on (TOCSY) total correlaion spectroscopy, 80 ms DIPSI2 mixing time) and (NOESY) nuclear overhauser enhancement spectroscopy, 300 ms NOE mixing time spectra. EDTA was added to the cofactor in a 1:1 ratio from a 0.5 M EDTA pH 7.5 stock.

##### Site‐Directed Mutagenesis

Site‐directed mutagenesis was performed on the pET17b_LmrR_M8D_K55D_K59Q_A92E template. Further (PCR) polymerase chain reaction and cloning were performed according to the methods described by Casilli et al.^[^ ^11^ ^]^ Primers are listed in Table S3, Supporting Information.

##### Synthesis of the Cofactors

The Cu(II)‐Phen cofactor was synthesized as previously described.^[^ ^7^ ^]^ As for the Zn(II)‐Phen cofactor, 100 mg of 1,10‐phenanthroline was mixed with 1.5 equivalents of ZnCl_2_ and dissolved in ethanol by stirring at 23 °C for 45 min. The suspension was filtered and the solid dried at room temperature. No further purification was needed. Elemental analysis calculated (%) for C_12_H_8_Cl_2_N_2_Zn: C, 45.54; H, 2.55; N, 8.85; measured (%): C, 45.48; H, 2.59; N, 8.71.

##### In Vitro Activity Assay

The biocatalytic FCA reaction was performed by first mixing 60 μM enzyme with 45 μM of Cu(II)‐Phen in a final volume of 290 μL of 20 mM MOPS and 150 mM NaCl, pH 7, and incubated at 4 °C for 30 min with gentle mixing. Afterwards, 1 mM of substrate S1 and S2a were added, bringing the final volume to 300 μL and 5 % of DMSO as cosolvent. The reaction mixture was incubated for 30 min at 4 °C with gentle mixing. Then 5 μL of internal standard (2‐phenylquinoline, 5 mM in CH_3_CN) was added and the reaction mixture was extracted with ethyl acetate (2 × 600 μL). The organic phase was dried with Na_2_SO_4_, filtered, and concentrated under reduced pressure. Subsequently, the sample was dissolved in 150 μL of a 9:1 mixture of n‐heptane and isopropyl alcohol. The yield and enantiomeric excess (ee) were determined by chiral (HPLC) high‐pressure liquid chromatography on a Chiralpak AD‐H column with 1 mL min^−1^ flow rate, using a 88:12 n‐heptane:isopropyl alcohol eluent composition and detected with (PDA) photodiode array at 254 nm. The calibration curve was obtained using a pure reference compound synthesized as in Chordia et al.^[^ ^7^ ^]^

## Conflict of Interest

The authors declare no conflict of interest.

## Supporting information

Supplementary Material

## Data Availability

The chemical shifts of WT and DE‐mutant LmrR are deposited in the Biological Magnetic Resonance Database (BMRB) under entries 52942 (WT) and 52943 (DE‐mutant).
