# Supplementary material for: Activity‐Enhancing Mutations in an LmrR‐Based Artificial Metalloenzyme Destabilize the Protein Scaffold and Alter its Conformational Plasticity
Source: Chembiochem. 2025 Jul 9;26(16):e202500259. doi: 10.1002/cbic.202500259 (PMC12432538; doi:10.1002/cbic.202500259)
Supplement: Supplementary file 1 — Supplementary Material [file CBIC-26-e202500259-s001.pdf]

Supplemental Information

for

Activity-enhancing mutations in an LmrR-based artificial metalloenzyme destabilize the protein scaffold and alter its conformational plasticity

A. A. Safeer[1], F. Casilli[2], J. W. Beugelink[3], G. Roelfes[2], M. Baldus[1], H. van Ingen[1,*]

[1] NMR Spectroscopy, Bijvoet Center for Biomolecular Research, Utrecht University, Padualaan 8, 3584 CH Utrecht, The Netherlands

[2] Biomolecular Chemistry & Catalysis, Stratingh Institute for Chemistry, University of Groningen, Nijenborgh 3, 9747 AG Groningen, The Netherlands

[3] Structural Biochemistry, Bijvoet Center for Biomolecular Research, Utrecht University, Universiteitsweg 99, 3584 CG Utrecht, The Netherlands

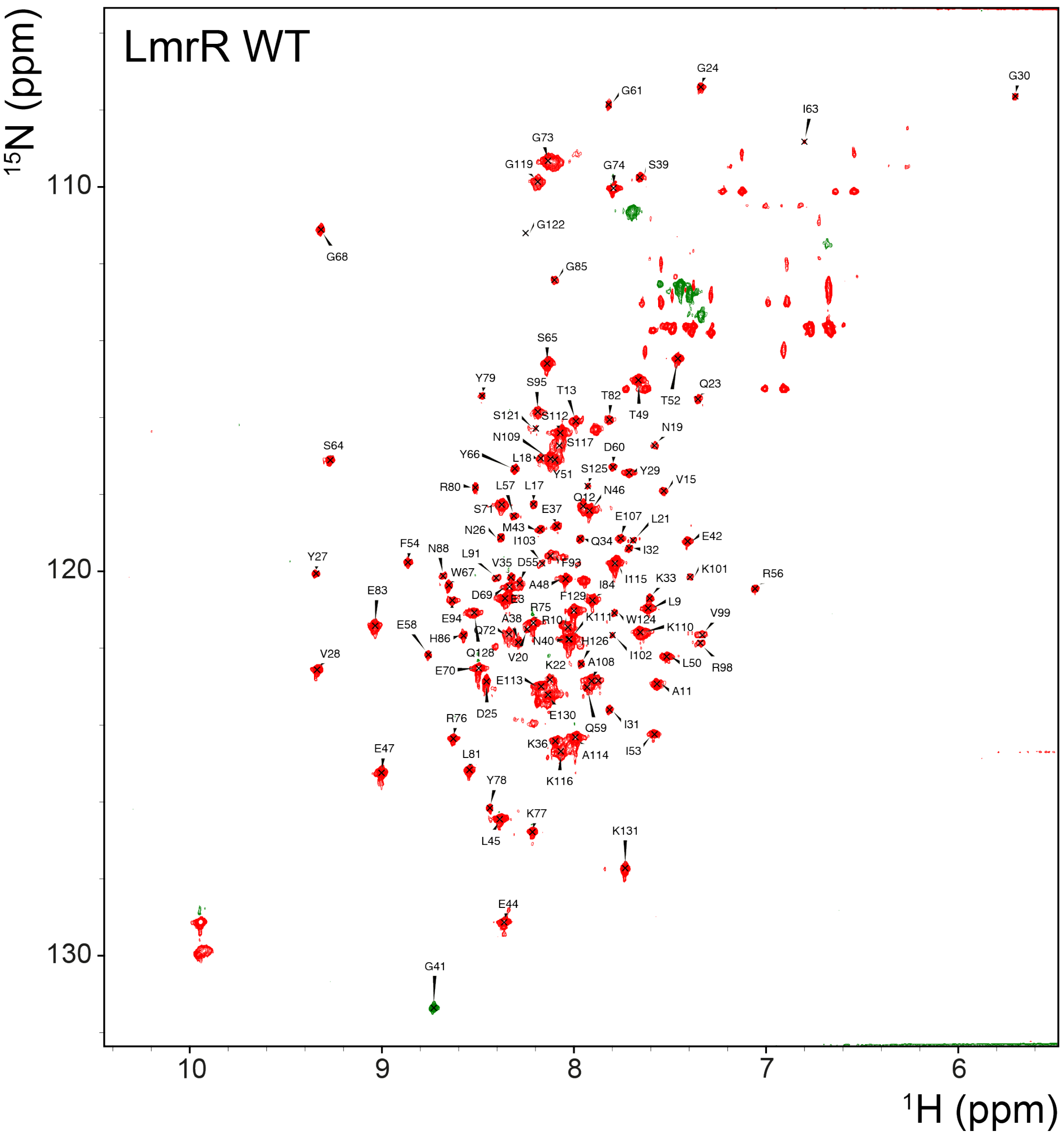

Figure S1. ^{15}N-HSQC spectrum of $^{2}H,^{15}N$-labeled LmrR WT (0.5 mM) in the apo condition.

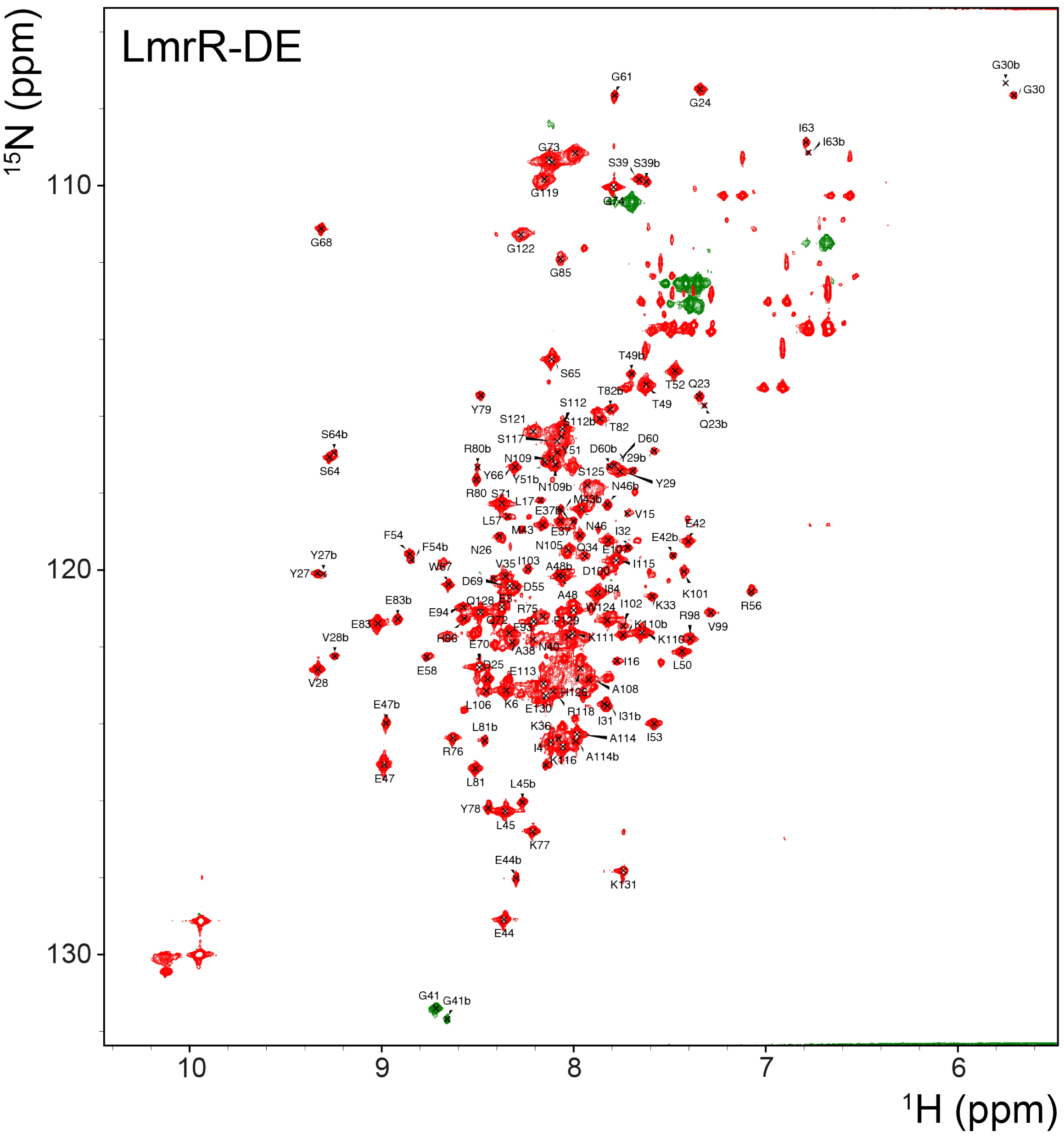

Figure S2. ^{15}N-HSQC spectrum of ^{2}H,^{15}N-labeled LmrR-DE (0.5 mM) in the apo condition. At high concentration the monomer peaks (labeled as 'b') are less pronounced.

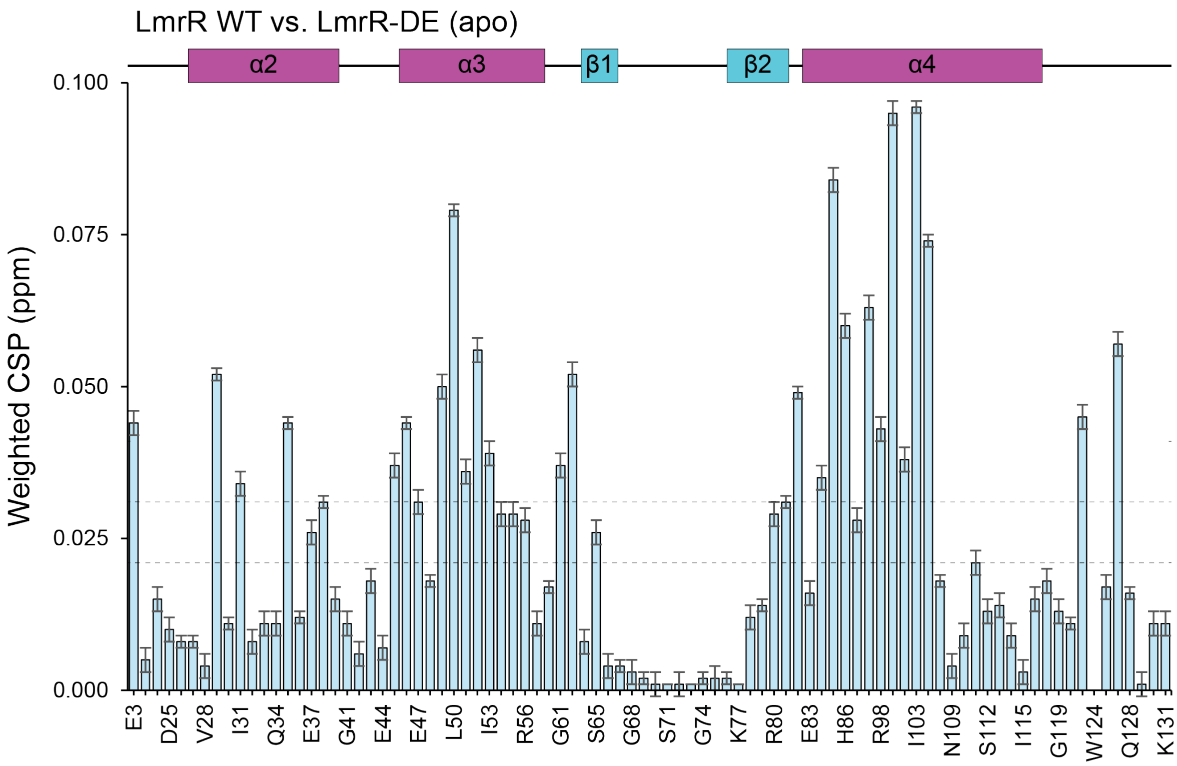

Figure S3. Backbone chemical shift perturbations for WT LmrR versus LmrR-DE resonance in the apo condition. Spectra were recorded at near equal protein concentration (~0.5 mM). Dashed lines (from low-to-high) indicate 10% trimmed mean, 1.5x 10% trimmed mean, and 2x 10% trimmed mean.

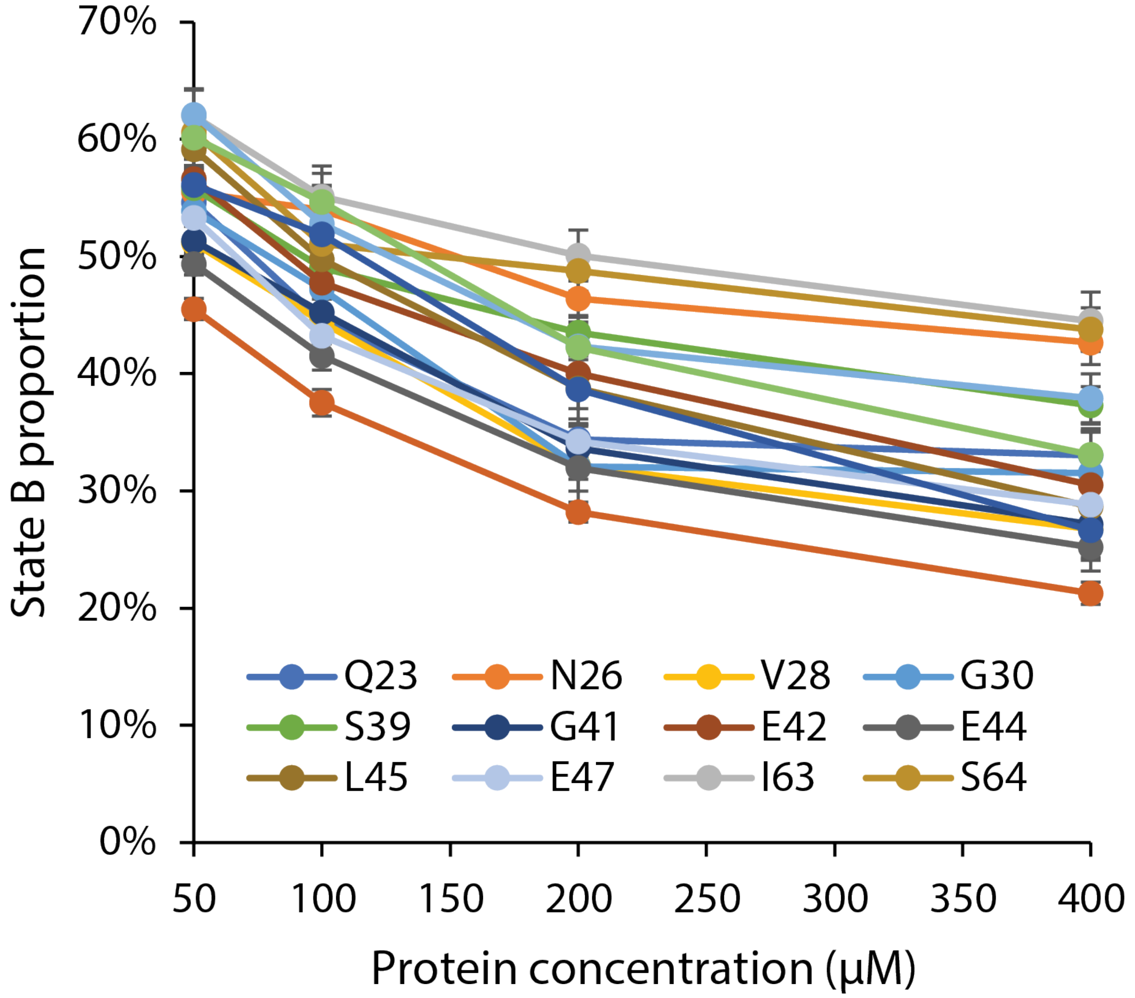

Figure S4. NMR-observed concentration-dependence of LmrR-DE state B proportion. Peak intensities were considered for 16 LmrR-DE residues with isolated monomer and dimer amide backbone peaks across a 50 – 400 μM (monomer) protein concentration series. Errors were based on the signal-to-noise level of the individual peaks.

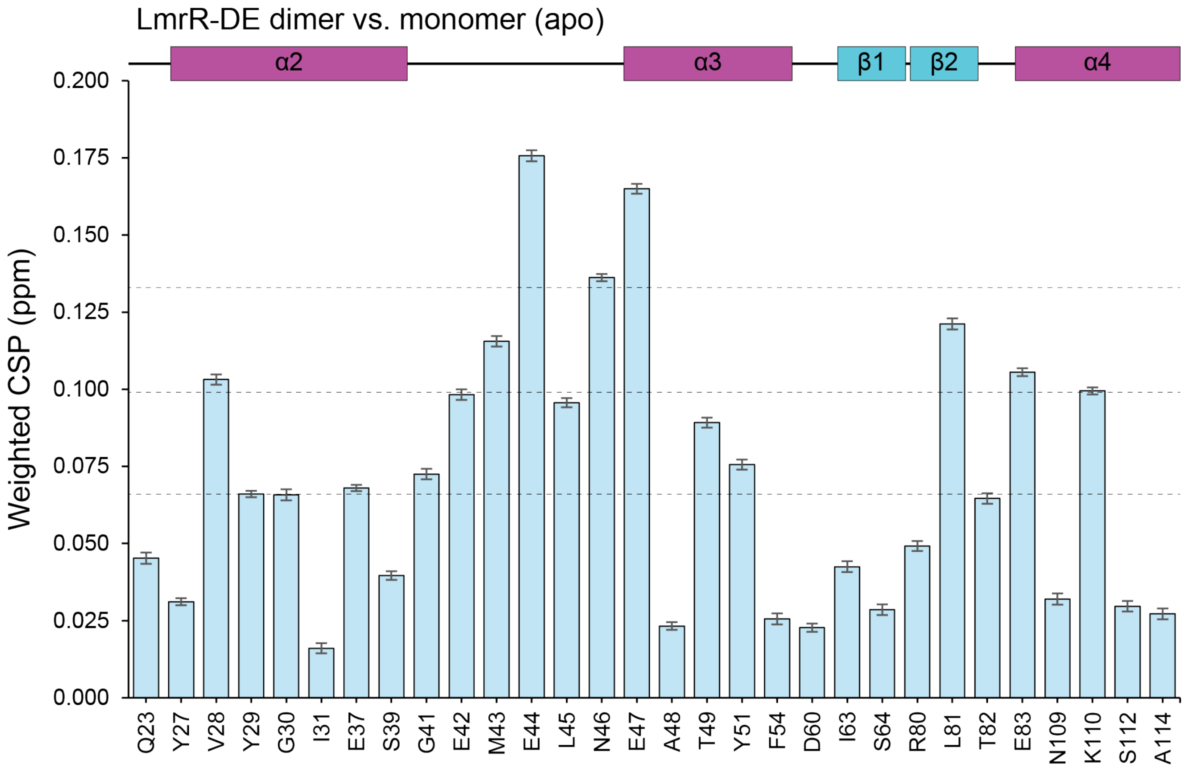

Figure S5. Backbone chemical shift perturbations for dimer versus monomer resonances of LmrR-DE in the apo condition. A ^{15}N-HSQC spectrum was recorded at 75 µM protein concentration to bring about sufficient monomer resonances. Dashed lines (from low-to-high) indicate 10% trimmed mean, 1.5x 10% trimmed mean, and 2x 10% trimmed mean.

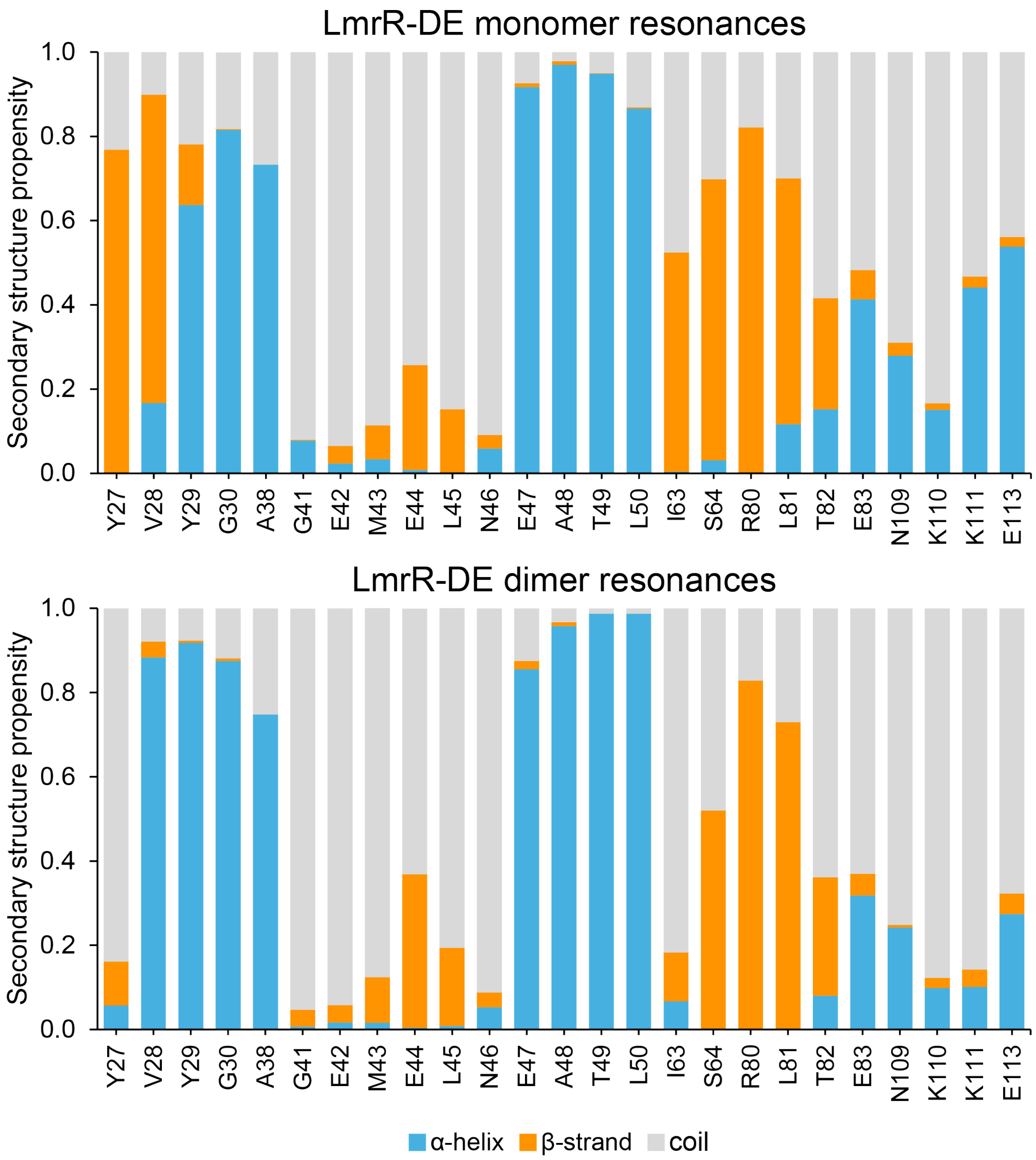

Figure S6. TALOS-N based secondary structure propensities. Predicted secondary structures based on monomer backbone chemical shifts (Cα and Cβ) align with those based on chemical shift values of dimer resonances, except for residues Tyr27, Val28 and Ile63.

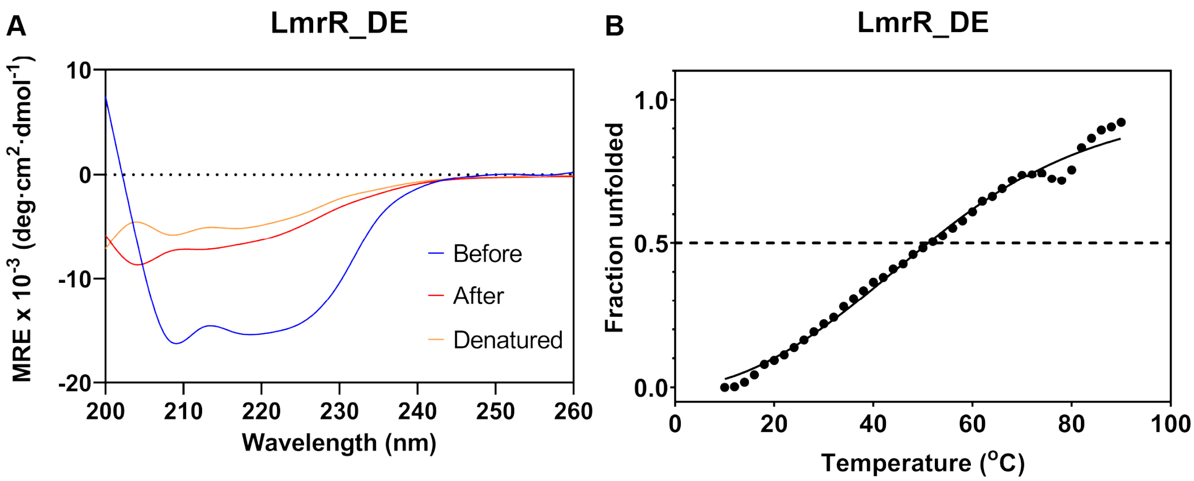

Figure S7. Thermostability of LmrR-DE. **A**) CD-spectra of LmrR-DE (blue) and after (red) a 10-90°C temperature series. Protein was denatured with heat treatment at 95°C for the reference spectrum. **B**) Melting temperature was determined at 50% protein in unfolded state.

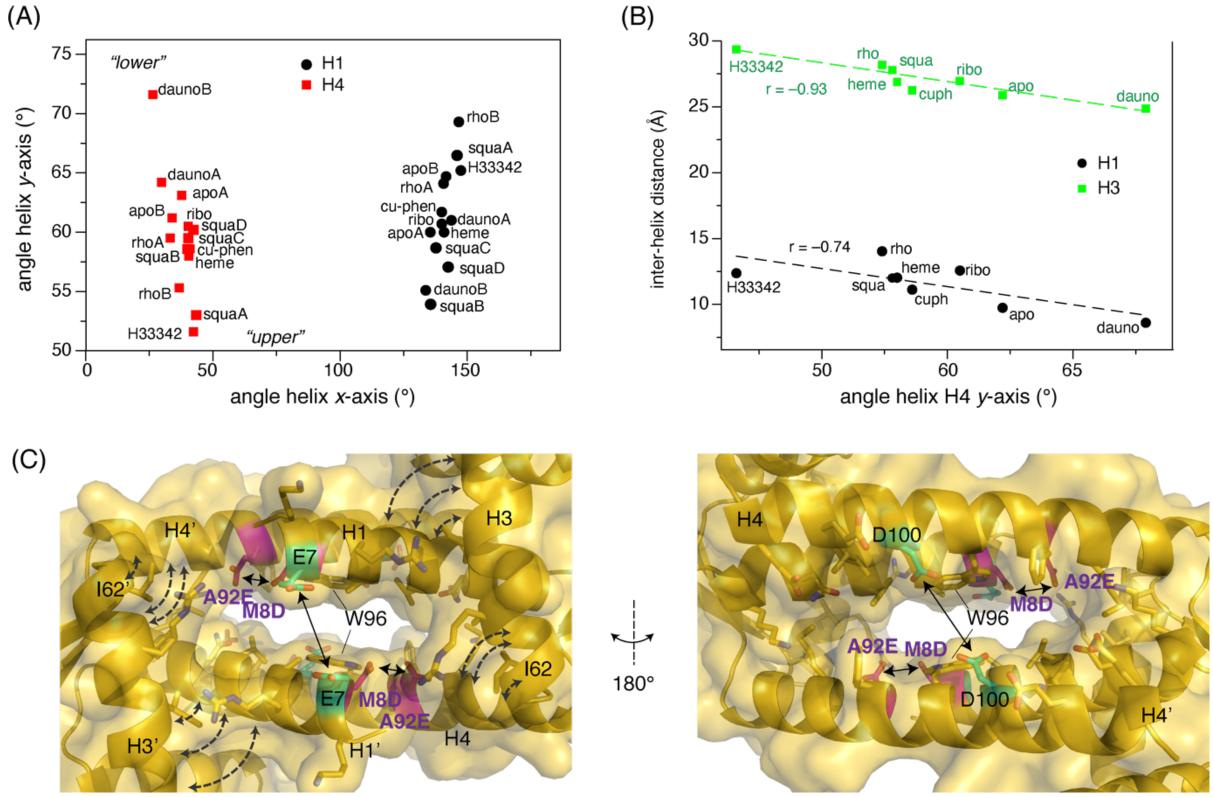

Figure S8. Allosteric coupling between orientations of helix H1, H3, and H4 and pocket dimensions. **A)** Analysis of helix H1 and H4 orientations across eight wildtype LmrR crystal structures (see Table S1) showing the angle with respect to *x*- (horizontal) and *y*- (vertical) axis of the fixed frame of Figure 1c in the Main Text. **B)** Correlation between helix H4 conformation and inter-helix distance. Correlation coefficients (*r*) indicated. Data points in A) & B) are labeled with the structure abbreviations shown in Table S1. **C)** Zoom-in of the hydrophobic pocket of apo LmrR (PDB: 3F8B) with modelled sidechains of M8D and A92E.The like-charge ion pairs formed by Glu7 in H1/H1' and Asp100 in H4/H4' are shown in green. The A92E and M8D mutations can be expected to generate a third instance of like-charge residues that point towards each other in the hydrophobic pocket. The molecular surface together with selected side chains in and around the pocket is shown. Helices and selected side chains are labeled. Dashed arrows indicate the potential interplay between helix packing that couples the pocket to the H4 conformation.

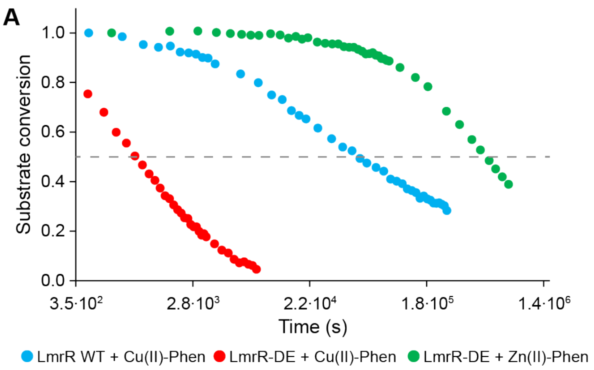

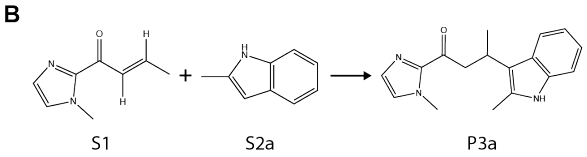

C

	Conversion $t_{1/2}$ (s)
LmrR WT + Cu(II)-Phen	$5.3 \cdot 10^4$
LmrR-DE + Cu(II)-Phen	$1.0 \cdot 10^3$
LmrR-DE + Zn(II)-Phen	$5.2 \cdot 10^5$

Figure S9. Reactivity analysis of LmrR for the FCA reaction tracked via NMR. **A)** Conversion of substrate under LmrR DE+Cu(II)-Phen (red, 18 μM), DE+Zn(II)-Phen (green, 20 μM) and WT+Cu(II)-Phen (blue, 20 μM) conditions. Normalized peak intensity is used to track conversion and the time (y) axis is displayed in log-scale. **B)** Reaction scheme of substrates 1 and 2a forming product 3a catalyzed by the LmrR-cofactor complexes. **C)** The estimated reaction half-times for substrate 2a conversion shows that the DE+Cu(II)-Phen complex is the most reactive condition.

To assess the FCA-activity of LmrR-DE+ZnPh, the LmrR-DE+CuPh, and the LmrR-WT+CuPh, 1D experiments were recorded at 298 K sample temperature on a Avance II 600 MHz Bruker spectrometer with a 5 mm liquid nitrogen-cooled cryo-probe. Substrate **S1** in reaction buffer and **S2a** in acetonitrile (both 1 mM from 125 mM stock solutions) were added to 20 μM of the assembled protein-cofactor complex in reaction buffer with 10% D_2O. Protonated protein was used to assemble all complexes.

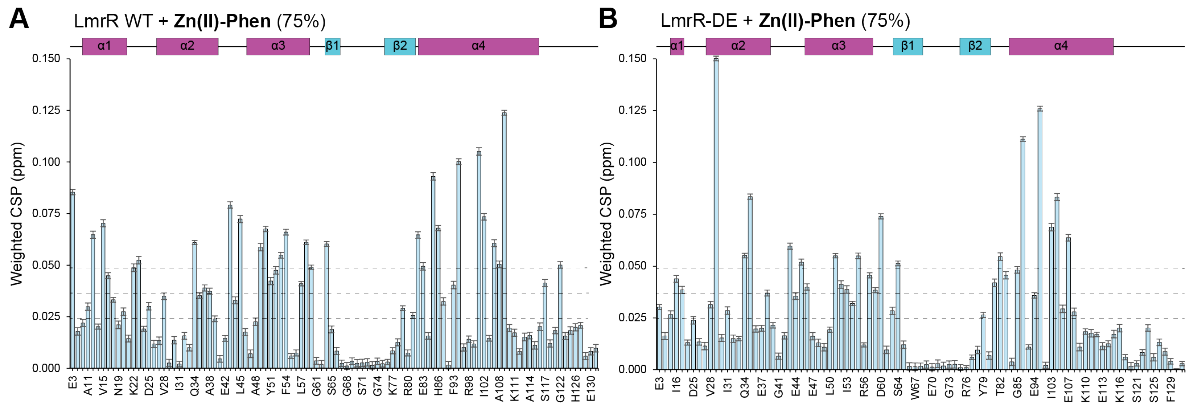

Figure S10. Backbone resonance chemical shift perturbations for cofactor addition to LmrR. A) CSPs upon addition of Zn(II)-Phen to 75 µM WT LmrR. **B)** CSPs upon addition of Zn(II)-Phen to 75 µM LmrR-DE. For both variants Zn(II)-Phen was added in a 1:1.5 ratio to the protein dimer (or 75% to all monomers) to achieve full occupation. Dashed lines (from low-to-high) indicate 10% trimmed mean, 1.5x 10% trimmed mean, and 2x 10% trimmed mean.

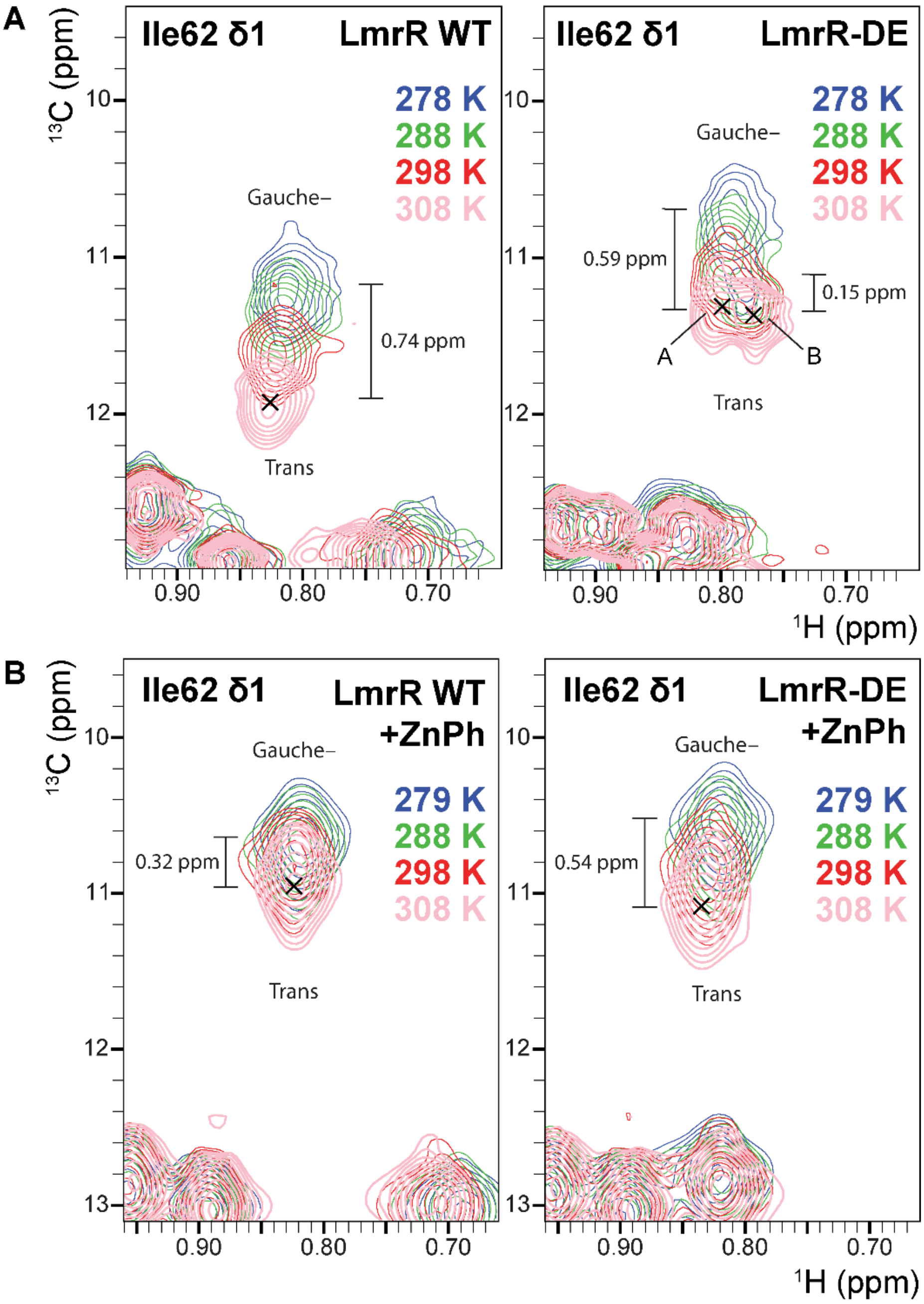

Figure S11. The dynamic equilibrium of the hydrophobic LmrR dimer pocket is heavily influenced by successive mutations. **A)** Methyl-TROSY cutout of Ile62 δ1 resonance for apo WT (left) and DE (right) variants at a 278 - 308 K temperature range. **B)** Methyl-TROSY cutout of Ile62 δ1 resonance for LmrR-WT (left) and LmrR-DE (right) bound with Zn(II)-Phen at a 279-308 K temperature range.

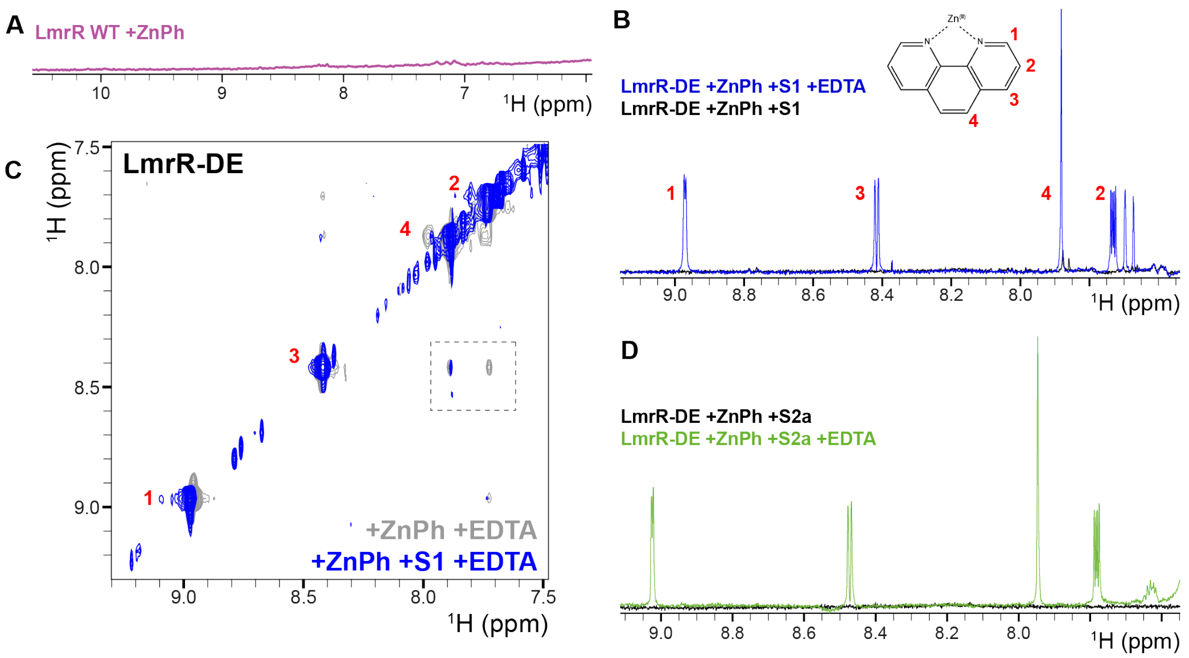

Figure S12. $^{13}C/^{15}N$-filtered spectra of EDTA supplementation to the protein complex in the presence of substrate. **A)** Zn(II)-Phen cofactor signals are not observed when bound to WT LmrR. **B) S1** presence upon EDTA-addition in a 1:1 ratio to LmrR-bound Zn(II)-Phen results in significant narrowing of linewidth for the recovered cofactor. **C)** Fewer NOESY cross-peaks (see box) appear for Zn(II)-Phen when recovered with **S1** indicates that in the absence of substrate the cofactor-EDTA complex still weakly binds to the protein and that **S1** can compete this effect away. **D)** The increase in resolution for cofactor peak also occurs in the presence of **S2a**.

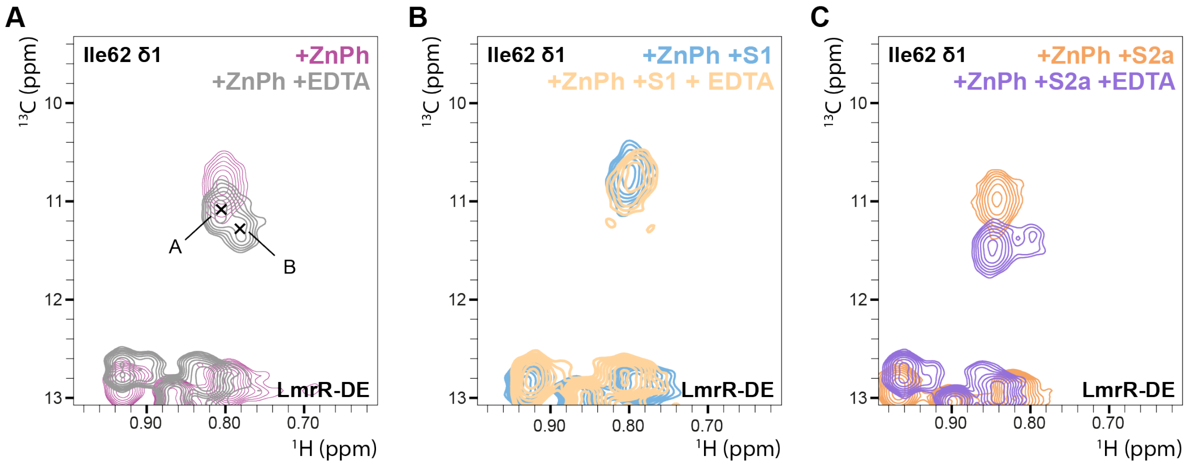

Figure S13. EDTA addition affects chemical shift of the Ile62 δ1 resonance. **A)** Methyl-TROSY cutouts of the LmrR-DE in complex with Zn(II)-Phen (purple). The resonance shifts to apo position and monomer population re-appears upon EDTA addition (grey) indicating cofactor release. **B)** Methyl-TROSY cutouts of the LmrR-DE in complex with Zn(II)-Phen in presence of 7.5 mM **S1** (light blue). The resonance does not shift to apo position nor does monomer population return upon EDTA addition (gold). **C)** Methyl-TROSY cutouts of the LmrR-DE in complex with Zn(II)-Phen in presence of 7.5 mM **S2a** (light orange). Addition of **S2a** induces a downfield ^{1}H peak shift as a result of the acetonitrile solvent. The resonance shifts downfield in the ^{13}C dimension upon EDTA addition (violet) and a monomer population seems to re-appear. For all samples, protein concentrations were 75 µM and EDTA was added in a 1:1 ratio to the cofactor.

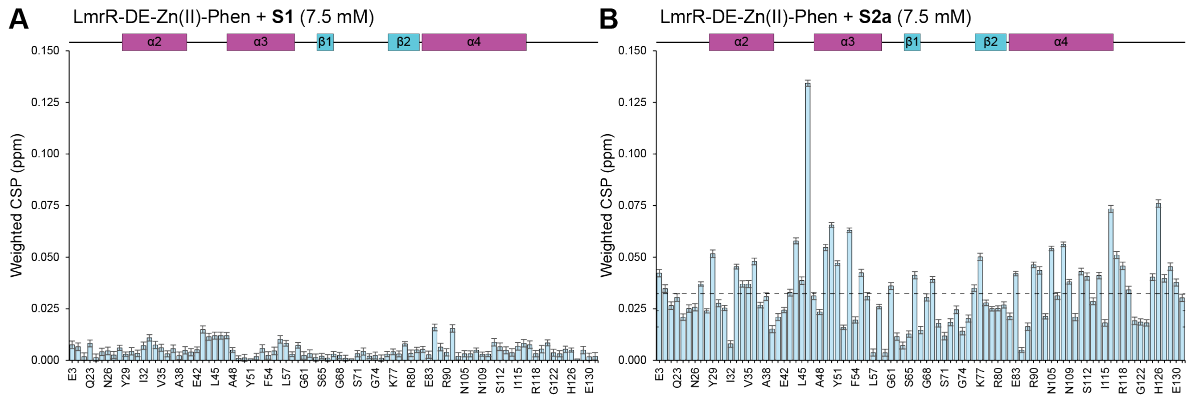

Figure S14. Backbone resonance chemical shift perturbations for substrate addition to the LmrR-cofactor complex. **A)** CSPs upon addition of **S1** (7.5 mM) to 75 µM LmrR-DE-Zn(II)-Phen. **B)** CSPs upon addition of **S2a** (7.5 mM) to 75 µM LmrR-DE-Zn(II)-Phen. For both variants Zn(II)-Phen was added in a 1:1.5 ratio (or 75% to monomers) to the protein to achieve full occupation. Dashed lines (from low-to-high) indicate 10% trimmed mean, 1.5x 10% trimmed mean, and 2x 10% trimmed mean.

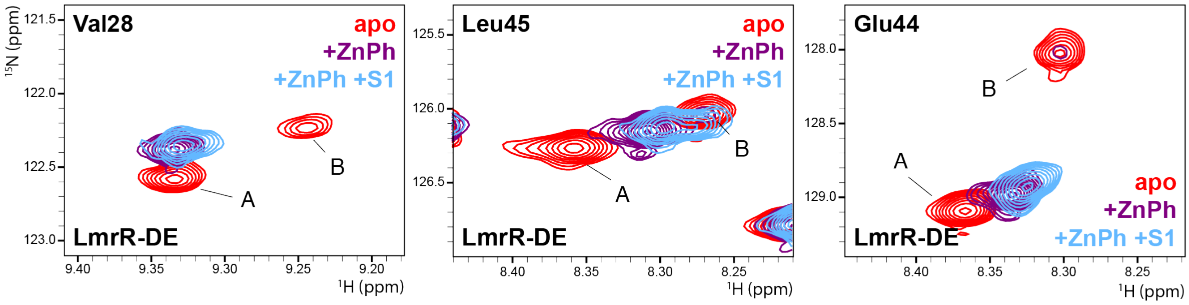

Figure S15. ^{15}N-HQSC cutouts of spectral changes upon S1 addition to LmrR. Backbone peaks of LmrR-DE shift upon addition of (7.5 mM) substrate **S1** to the protein-cofactor complex and an additional monomer-like (Leu45) state manifests in slow exchange.

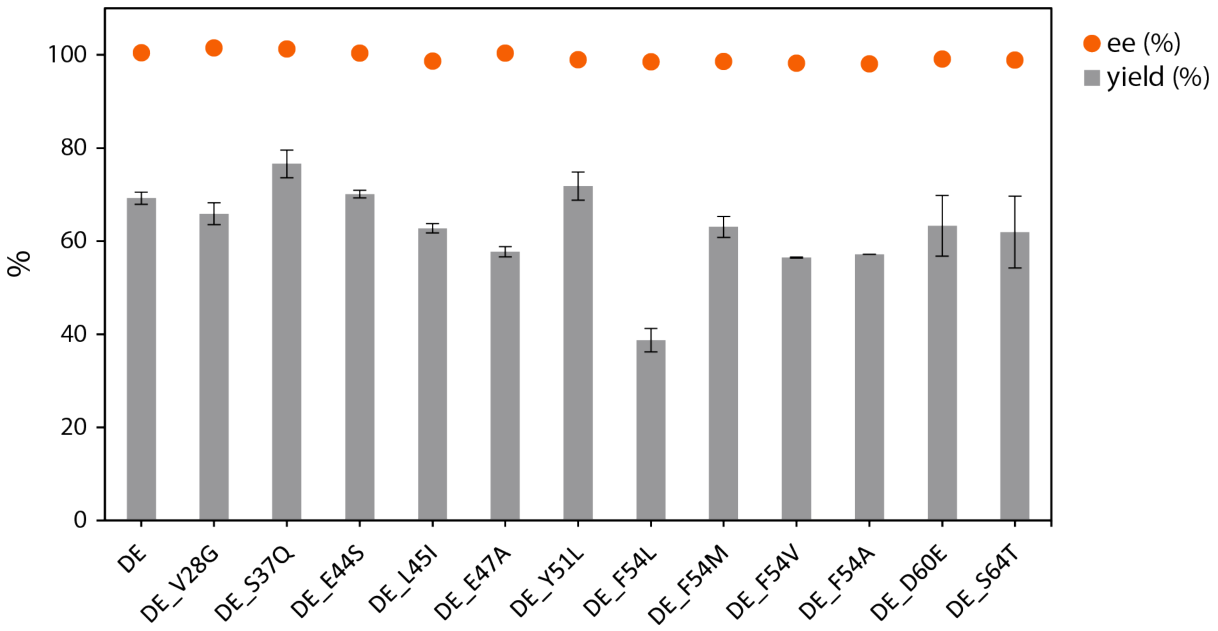

Figure S16. FCA-activity of the screened LmrR-DE mutants. Yield and enantioselectivity (ee) were assessed by HPLC analysis of the product formed by the reaction between substrates **S1** and **S2a**. Typical reaction conditions were 1.5 mM of **S1** and 1 mM of **S2a** with 20 μM of protein in the reaction buffer. The error bars represent the standard deviation of at least three independent experiments.

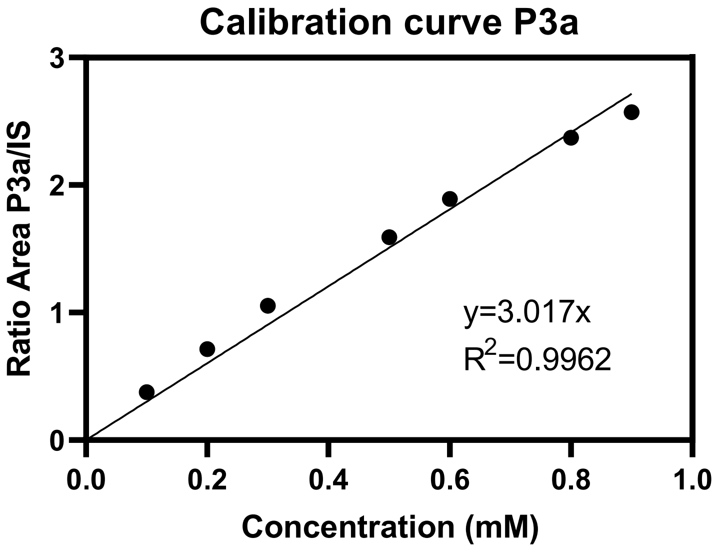

Figure S17. Calibration curve used to determine yield of product **P3a** upon FCA between substrates **S1** and **S2a**. IS: Internal Standard

Table S1: Structural analysis of LmrR crystal structures

PDB	year	description (bound ligand)	Ile62 χ_2 angle (°) (chain A/B)	angle H1 y-axis[a] (°)	angle H4 y-axis[a] (°)	upper / lower[b]	distance H3-H3'[c] (Å)	distance H1-H1'[d] (Å)
3F8C	2009[19]	Hoechst 33342 (H33342)	-59.6 (g–)	65.2	51.7	upper	29.4	12.4
6DO0	2018[23]	rhodium bis-phosphine (rho)	-58.6 (g–) /-165.1 (t)	64.1 69.3	59.5 55.3	upper	28.2	14.0
6FUU	2020[26]	heme	-51.6 (g–)	60.0	58.0	upper	26.9	12.0
6R1L	2019[8]	Cu(II)-phenanthroline (cuphen)	-63.5 (g–)	61.7	58.6	upper	26.3	11.1
4ZZD	2015[22]	riboflavin (ribo)	-45 (g–)	60.7	60.5	upper	27.0	12.6
3F8B	2009[19]	apo	-64.0 (g–) /-58.9 (g–)	60.0 64.7	63.1 61.2	lower	25.9	9.7
3F8F	2009[19]	daunomycin (dauno)	154.9 (t) / 157.2 (t)	61.0 55.1	64.2 71.6	lower	24.9	8.6
7Q34	2022(ref)	squaraine (squa)	-66.2 (g–) / -74.9 (g–) / -42.6 (g-) / -62.4 (g-)	66.3 / 58.6 53.9 / 57.0	53.0 / 58.5 59.4 / 60.1	upper	27.9 / 27.9	12.6 / 11.4

[a] angle of helix-axis with respect to the vertical axis (*y*-axis) in the orientation of Figure 5.1C.
[b] classification of the helix H4 conformation based on the angle of the H4 helix axis with respect to the *y*-axis, > 61° is "lower" and <= 61° is "upper".
[c] measured between CA atoms of Arg56
[d] measured between CA atoms of Glu7

Table S2. LmrR-DE monomer population.

NMR[a]			SEC-MALS	
Conc (μM)	Conc (mg/ml)	Monomer (%)	Conc (mg/ml)	Monomer (%)
400	6.0	32 ± 7	8.0	40 ± 3
200	3.0	39 ± 7	4.0	51 ± 3
100	1.5	48 ± 5	2.0	61 ± 2
50	0.8	55 ± 5	1.0	73 ± 2
			0.5	80 ± 3

[a] Uncertainty for NMR data is the standard deviation for 16 residues with isolated monomer and dimer backbone peaks.

Table S3. Primers used in site-directed mutagenesis for directed evolution. The forward (fwd) and reverse (rv) primers for the introduction of each mutation are reported. The mutated codons are shown in small letters.

Primer name	Sequence (3'->5')
V28G_fwd	GCGATAACTATggtTATGGCATTATCAAACAGGTG
V28G_rv	ATGCCATAaccATAGTTATCGCCTTGTTTCAGG
E37Q_fwd	CAGGTGAAAcagGCGAGCAACGGTG
E37Q_rv	TTGCTCGCctgTTTCACCTGTTTGATAATGC
E44S_fwd	CGGTGAAATGagcCTGAATGAAGCCACCCTGT
E44S_rv	CTTCATTCAGgctCATTTCACCGTTGCTCGC
L45I_fwd	AAATGGAAattAATGAAGCCACCCTGTATACG
L45I_rv	GGCTTCATTaatTTCCATTTCACCGTTGC
E47A_fwd	GAACTGAATgcgGCCACCCTGTATACGATTC
E47A_rv	AGGGTGGCcgcATTCAGTTCCATTTCAC
Y51L_fwd	CACCCTGctgACGATTTTTGATCGTCTGGAAC
Y51L_rv	AAAATCGTcagCAGGGTGGCTTCATTCAGTTC
F54L_fwd	TATACGATTctgGATCGTCTGGAACAGGACGG
F54L_rv	CCAGACGATCcagAATCGTATACAGGGTGG
F54M_fwd	TATACGATTatgGATCGTCTGGAACAGGACG
F54M_rv	CCAGACGATCcatAATCGTATACAGGGTGG
F54V_fwd	TATACGATTgtgGATCGTCTGGAACAGGACG
F54V_rv	CCAGACGATCcacAATCGTATACAGGGTGG
F54A_fwd	TATACGATTgcgGATCGTCTGGAACAGGACGG
F54A_rv	CCAGACGATCcgcAATCGTATACAGGGTGG
D60E_fwd	GAACAGgaaGGCATTATCAGCTCTTAC
D60E_rv	TAATGCCttcCTGTTCCAGACGATCAA
S64T_fwd	GGCATTATCaccTCTTACTGGGGTGATGA
S64T_rv	CAGTAAGAggtGATAATGCCGTCC
